# Silica sulfuric acid: a reusable solid catalyst for one pot synthesis of densely substituted pyrrole-fused isocoumarins under solvent-free conditions

**DOI:** 10.3762/bjoc.9.269

**Published:** 2013-11-04

**Authors:** Sudipta Pathak, Kamalesh Debnath, Animesh Pramanik

**Affiliations:** 1 Department of Chemistry, University of Calcutta, 92, A. P. C. Road, Kolkata-700 009, India; Fax: +91-33-2351-9755; Tel: +91-33-2484-1647

**Keywords:** green chemistry, pyrrole-fused isocoumarin, reusable solid support, silica sulfuric acid, solvent-free condition

## Abstract

A convenient and efficient methodology for the synthesis of densely substituted pyrrole-fused isocoumarins, which employs solid-supported silica sulfuric acid (SSA) as catalyst, has been developed. When the mixture of ninhydrin adducts of acetylacetone/ethyl acetoacetate and primary amines was heated on the solid surface of SSA under solvent-free conditions, the pyrrole-fused isocoumarins were formed in good yields. This synthetic method has several advantages such as the employment of solvent-free reaction conditions without the use of any toxic reagents and metal catalysts, the ease of product isolation, the use of a recyclable catalyst, the low cost, the easy availability of the starting materials, and the excellent yields of products.

## Introduction

Isocoumarins are an important class of naturally occurring lactones [1–3], which has attracted the attention of chemists because of their various biological activities such as antioxidative [4], anticancer [5] and antifungal activities [6]. The development of a new and efficient methodology for the synthesis of biologically potent isocoumarins and their carbo/hetero annulated analogues has drawn great attention of synthetic as well as medicinal chemists [7–9]. Various methodologies for the synthesis of isocoumarins have been reported such as the reaction of *o*-halobenzoic acids and 1,3-diketones through a copper-catalyzed tandem sequential cyclization/addition/deacylation process [10–11], an iridium-catalyzed oxidative lactonization or an intramolecular cyclization reaction of δ-ketoaldehydes [12], a ruthenium-catalyzed aerobic oxidative cyclization of aromatic acids with alkynes [13], an FeCl_3_-promoted regioselective annulation of *o*-(1-alkynyl)benzoates with disulfides [14], a Heck–Matsuda cyclization reaction [15], a 6-*endo-dig* cyclization of heteroaryl esters to alkynes [16], or a Pd(II)-mediated cyclization of *o*-allylbenzaldehydes [17]. Salvinorin A, a natural product isolated from the hallucinogenic sage *Salvia divinorum*, which also contains a saturated isocoumarin ring, has been synthesized [18]. Although these methods are useful for the synthesis of isocoumarin derivatives, the reactions involved in the synthesis still suffer from some serious limitations such as the use of expensive and hazardous reagents [12] and toxic metal catalysts [10–11,15,17]. Some of the reactions need laborious and time consuming procedures [12–13,17], or drastic reaction conditions and with only low to moderate yields [16]. On the other hand, although a number of synthetic methods have been developed for the construction of densely substituted pyrrole rings [19–21], not a single report has been given on the synthesis of pyrrole-fused isocoumarins with the help of green methodology, so far. Therefore, the development of an environmentally friendly and safer reaction methodology following the green chemistry principles is essential for the synthesis of pyrrole-fused isocoumarins.

**Scheme 1:**
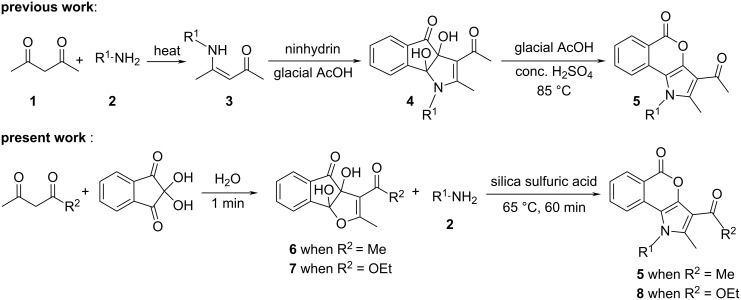
Synthesis of pyrrole-fused isocoumarins.

The employment of a reusable solid supported/heterogeneous catalyst for the efficient synthesis of heterocyclic compounds remains a challenge to chemists in laboratories and in the industry [22–23]. Reactions with reagents that are immobilized on inorganic solid supports show several advantages over the conventional reactions in solution because of simple work-up procedures, improved product yields, greater ease of purification, shorter reaction times, milder reaction conditions, and recyclability of the catalyst [24]. In the recent years, silica sulfuric acid (SSA) has shown immense potentiality as an efficient and easily retrievable solid catalyst in various important organic syntheses under solvent-free conditions [25]. The high catalytic activity, the operational simplicity and the recyclability of SSA can be exploited in the industry for the synthesis of various drugs and pharmaceuticals. SSA, a product that is easily synthesized from silica gel and chlorosulfonic acid [26], was observed to improve the reactivity and selectivity in carbon–carbon bond-formation reactions [27–28], in cycloaddition reactions [29–30], in protection–deprotection reactions of multistep syntheses [31–33], in esterifications [34] and in syntheses of heterocycles [35]. Since we are actively involved in the synthesis of biologically important heterocycles [36–42], we wish to report herein a green methodology for the construction of pyrrole-fused isocoumarins, which uses SSA as a solid-supported acid catalyst under solvent-free conditions (Scheme 1, present work).

## Results and Discussion

Recently, we have reported that the enamines **3** generated from acetylacetone (**1**) and amines **2** react with ninhydrin to form the cyclic hemiaminal dihydroxyindenopyrroles **4**. Subsequently intermediates **4** produce the pyrrole-fused isocoumarins **5** upon heating in glacial acetic acid with a catalytic amount of conc. H_2_SO_4_ (Scheme 1, previous work) [38]. It was observed that in the above synthesis the intermediate dihydroxyindenopyrroles **4** were needed to be isolated for further reaction to get the final products **5** in pure form. Otherwise some acetylated amines were always produced as byproducts. Besides, the formation of **4** from **3** did not proceed significantly when the enamines of ethyl acetoacetate were employed, because under acidic conditions, the enamines of ethyl acetoacetate readily hydrolyze and the free amines react with ninhydrin to form Schiff bases. To overcome the above problems we have designed an operationally simple one-pot reaction for the synthesis of pyrrole-fused isocoumarins (**5** or **8**) from the ninhydrin adducts of acetylacetone/ethyl acetoacetate (**6** or **7**) [43] and primary amines **2** under solvent-free conditions (Scheme 1, present work).

**Scheme 2:**
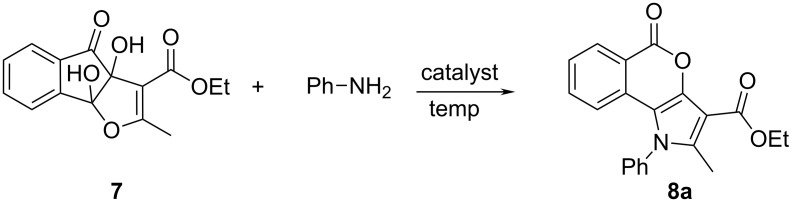
Reaction scheme for the synthesis of pyrrole-fused isocoumarins.

In order to explore the role of the different catalysts and solvents in the preparation of pyrrole-fused isocoumarins, an optimisation study was carried out with the model reaction between dihydroxyindenofuran ethyl ester **7** [43] and aniline in a molar ration of 1.00:1.50 (Scheme 2). When the reaction was carried out in aqueous solution under reflux the reaction did not proceed at all (Table 1, entry 1). Previous results showed that an activation by a Brønsted acid was necessary to carry out the reaction successfully [38]. Therefore, we screened various Brønsted acid catalysts, e.g., lactic acid, formic acid, citric acid and acetic acid in aqueous solution under reflux. But the yields were very low even after prolonged reaction time (Table 1, entries 2–5). On the basis of the assumption that more acidic conditions might be necessary to furnish the desired products in high yields, we carried out the reaction in acetic acid with adding a catalytic amount of H_2_SO_4_. Intriguingly, the yield of the product increased from less than 10% to 64% (Table 1, entry 6). The structure of the product **8a** was confirmed by IR, ^1^H NMR and ^13^C NMR spectroscopy and elemental analysis. Surprisingly, when the above-mentioned reaction was carried out with aliphatic amines, only the acetylated amines were obtained instead of the desired products **8**. These results influenced us to carry out the reaction under greener and milder reaction conditions, but with satisfying yield of the desired product, both for aromatic and aliphatic amines. We restrained the reaction to using PEG–OSO_3_H as a Brønsted acid–surfactant combined catalyst in aqeous solution under refluxing conditions as well as under solvent-free conditions (Table 1, entries 7 and 8). Although under solvent-free conditions the required temperature was lower and the yields of the products were higher, the yields were still only moderate. This encouraged us to execute the optimization study in presence of a solid acid catalyst under solvent-free conditions. This is one important facet of green chemistry: the eradication of solvents in chemical processes. Hence, we have carried out the synthesis by dissolving the substrate **7** and aniline in a minimum volume of chloroform, soaked them on the solid surface of solid Brønsted acid catalysts, such as silica gel and melamine sulfonic acid (MSA), dried the mixture under vacuum, and heated the reaction mixture to 100 °C (Table 1, entries 9 and 10). Unfortunately, the reactions on silica gel and MSA failed to give the desired product **8a**. In the search of a suitable solid acid catalyst we employed silica sulfuric acid (SSA) at 100 °C. However, the reaction mixture got charred after 0.5 h and a considerable amount of impurities along with the desired product **8a** was formed (Table 1, entry 11). When lowering the reaction temperature (65–100 ºC) and varying the amount (300–500 mg) of solid catalyst (Table 1, entries 12–15), the maximum yield (90%) of **8a** was obtained at 65 °C using 400 mg of SSA (Table 1, entry 14).

**Table 1:** Optimization of reaction conditions for the synthesis of **8a**.

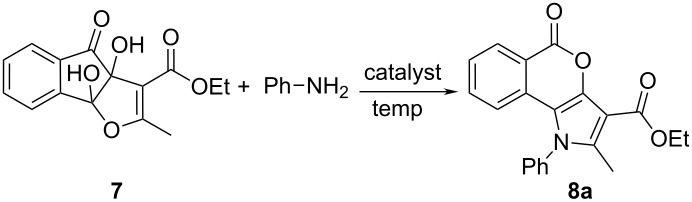						

entry	catalyst	solvent	catalyst load	temperature (°C)	time (h)	yield (%)^a^

1	—	H_2_O	—	100	24	—
2	lactic acid	H_2_O	20 mol %	100	24	5
3	formic acid	H_2_O	20 mol %	100	24	8
4	citric acid	H_2_O	20 mol %	100	24	5
5	acetic acid	H_2_O	20 mol %	100	24	6
6	H_2_SO_4_	acetic acid	20 mol %	85	1	64
7	PEG–OSO_3_H	H_2_O	500 mg	100	2	45
8	PEG–OSO_3_H	—	500 mg	80	1.5	66
9	silica gel	—	500 mg	100	24	—
10	melamine sulphonic acid	—	500 mg	100	24	—
11	silica sulfuric acid	—	500 mg	100	0.5	45
12	silica sulfuric acid	—	500 mg	85	0.5	58
13	silica sulfuric acid	—	500 mg	65	1	90
**14**	**silica sulfuric acid**	—	**400 mg**	**65**	**1**	**90**
15	silica sulfuric acid	—	300 mg	65	1.5	83

^a^Optimization studies were carried out with 1.0 mmol **7** and 1.5 mmol of aniline.

**Figure 1:**
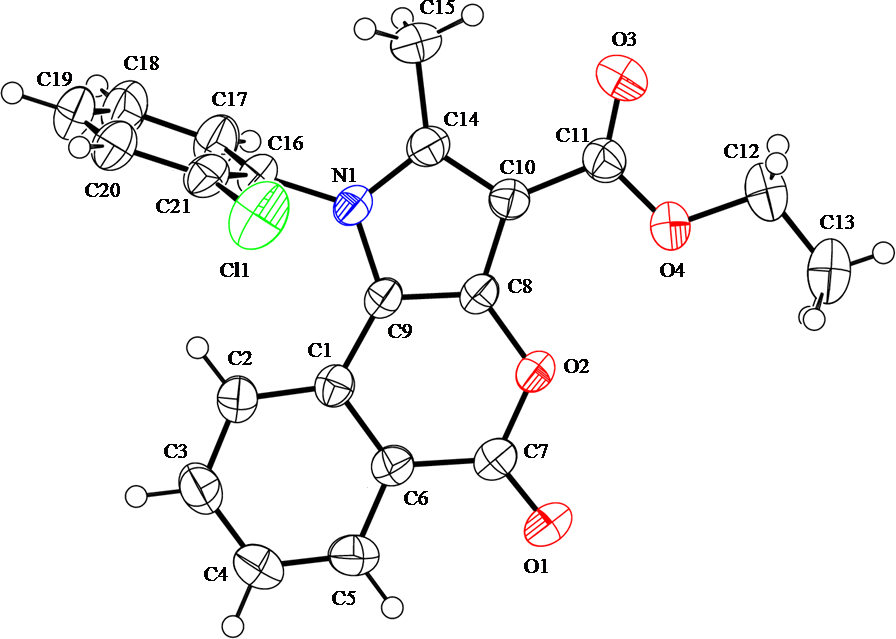
ORTEP diagram of **8c** with atom numbering scheme. Thermal ellipsoids are shown at 50% probability with CCDC number 949317.

Having successfully prepared **8a**, we decided to explore the scope and generality of this reaction in the synthesis of other analogues. Accordingly, the ninhydrin adducts of acetylacetone/ethyl acetoacetate (**6** and **7**) [43] were reacted with a variety of commercially available aliphatic and aromatic primary amines under the optimized conditions (Table 1, entry 14). As becomes evident from Table 2, all the primary amines reacted well with adducts **6** and **7** affording the desired products **5** and **8** in good yields. The results show that solvent-free conditions and the SSA catalyst are crucial carrying out the reaction succesfully even with aliphatic amines. The structures of the new products **8a**–**o** were determined by using spectroscopic data and elemental analysis. X-ray crystal data analysis of compound **8c** further confirmed the product formation (Figure 1). The formation of products **5a**–**l** was confirmed by comparing the reported spectral data and melting points (Table 2) [38].

**Table 2:** Formation of isocoumarins **5** and **8** from adducts **6** and **7** respectively on an SSA surface.

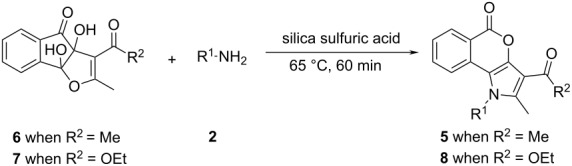						

entry	R^1^	R^2^	adduct	product	yield (%)^a^	mp observed/lit. [38] (°C)

1	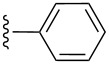	Me	**6**	**5a**	91	248–250/248
2	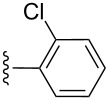	Me	**6**	**5b**	82	205–207/205
3	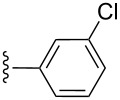	Me	**6**	**5c**	88	262–264/262
4	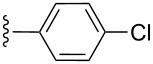	Me	**6**	**5d**	89	258–260/258
5	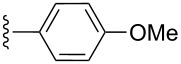	Me	**6**	**5e**	84	220–222/220
6	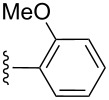	Me	**6**	**5f**	80	172–174/172
7	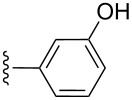	Me	**6**	**5g**	79	236–238/236
8	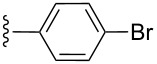	Me	**6**	**5h**	82	260–262/260
9	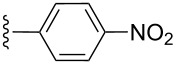	Me	**6**	**5i**	86	>320/>320
10	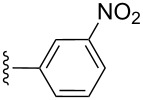	Me	**6**	**5j**	84	>320/>320
11	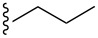	Me	**6**	**5k**	90	150–152/150
12	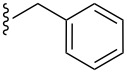	Me	**6**	**5l**	88	182–184/182
13	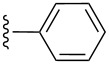	OEt	**7**	**8a**	90	208–210
14	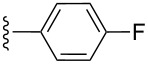	OEt	**7**	**8b**	89	252–254
15	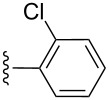	OEt	**7**	**8c**	79	233–235
16	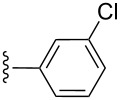	OEt	**7**	**8d**	85	230–232
17	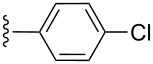	OEt	**7**	**8e**	87	218–220
18	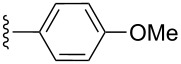	OEt	**7**	**8f**	80	194–196
19	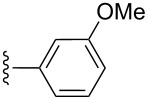	OEt	**7**	**8g**	83	198–200
20	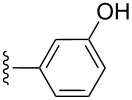	OEt	**7**	**8h**	81	254–256
21	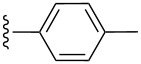	OEt	**7**	**8i**	86	202–204
22	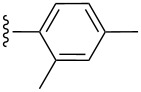	OEt	**7**	**8j**	81	190–192
23	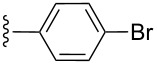	OEt	**7**	**8k**	83	212–214
24	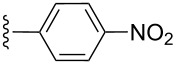	OEt	**7**	**8l**	86	260–262
25	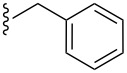	OEt	**7**	**8m**	91	180–182
26	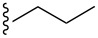	OEt	**7**	**8n**	89	132–134
27	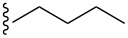	OEt	**7**	**8o**	87	125–127

^a^Isolated yield.

The comparative studies in terms of overall reaction times and product yields show that although the present method takes more time than the previous method [38], the overall yields of the products are larger in the present method (Table 3). More importantly the present method enables us to access a new series of pyrrole-fused isocoumarins with ester functionality (Table 3, compounds **8a–o**), which were not possible to synthesize with the previous method. Apart from that the present method is more advantageous in terms of product formation and greener characteristics than the previous one in many respects such as (a) a less laborious and more step-economical reaction for the library synthesis of pyrrole-fused isocoumarin derivatives, since the starting materials dihydroxy indenofurans **6** and **7** need only one step for preparation, (b) the employment of milder acidic conditions, (c) a lower reaction temperature (65 °C), (d) solvent-free conditions, and (e) more cost-effective because of the reusability of the solid-supported SSA. Moreover the starting materials **6** and **7** can also be prepared through a green methodology [43].

**Table 3:** Comparison between the present and the previous method for the synthesis of pyrrole-fused isocoumarins from ninhydrin.

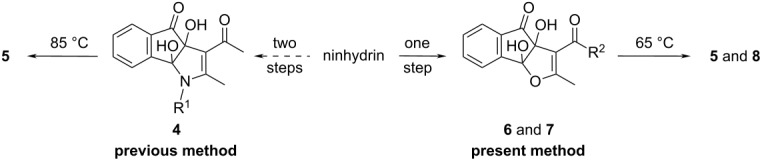					

entry	product	overall reaction time (min)		overall yield (%)	
		present method	previous method	present method	previous method

1	**5a**	61	37	87	81
2	**5b**	61	46	79	64
3	**5c**	61	45	84	74
4	**5d**	61	37	85	80
5	**5e**	61	39	81	80
6	**5f**	61	51	77	66
7	**5g**	61	37	76	58
8	**5h**	61	36	79	77
9	**5i**	61	37	83	56
10	**5j**	61	46	81	68
11	**5k**	61	21	86	78
12	**5l**	61	25	84	84
13	**8a**	61	—	84	—
14	**8b**	61	—	83	—
15	**8c**	61	—	73	—
16	**8d**	61	—	79	—
17	**8e**	61	—	81	—
18	**8f**	61	—	74	—
19	**8g**	61	—	77	—
20	**8h**	61	—	75	—
21	**8i**	61	—	80	—
22	**8j**	61	—	75	—
23	**8k**	61	—	77	—
24	**8l**	61	—	80	—
25	**8m**	61	—	85	—
26	**8n**	61	—	83	—
27	**8o**	61	—	81	—

**Scheme 3:**
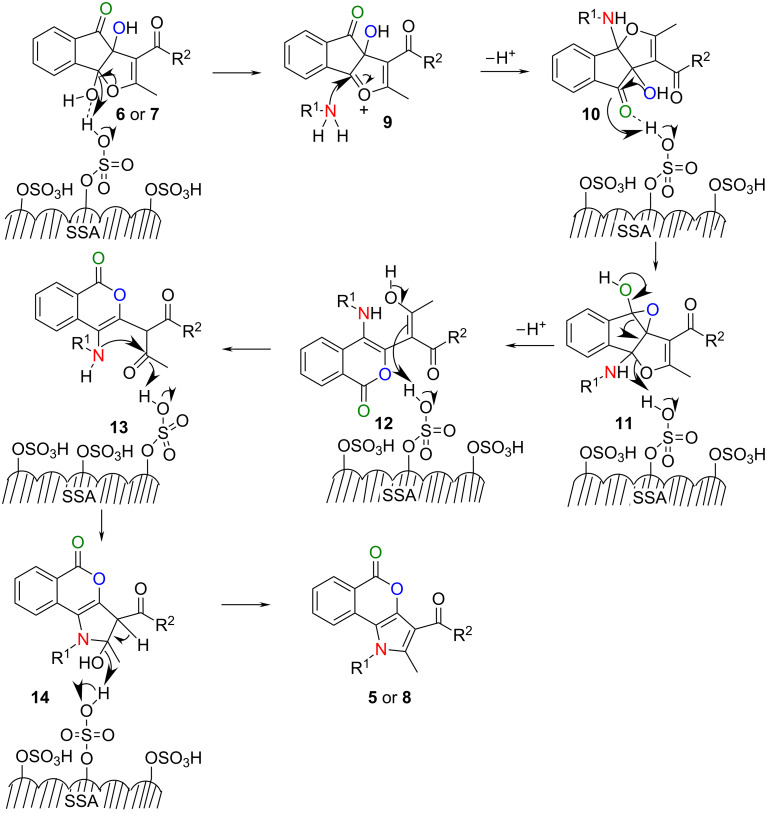
Mechanism of formation of isocoumarins **5** or **8** on the surface of SSA.

Based on the results of Table 2 and the fact that SSA plays the role of transferring protons from its solid surface, a probable mechanism for the formation of isocoumarins **5** or **8** is explicated in Scheme 3. The protonation and activation of the hydroxy group of dihydroxy indenofuran (**6** or **7**) by the sulfonic group of SSA generates dehydrated cationic intermediate **9**. This reactive intermediate **9** provokes a nucleophilic attack of primary amines to form bicyclo[3.3.0]octanamino compound **10**. Then the α-hydroxy group of **10** attacks the adjacent carbonyl carbon to generate epoxy intermediate **11**. This unstable epoxy intermediate **11** produces a six-membered lactone intermediate **12** through the breaking of a C–C bond. Subsequently, intermediate **12** tautomerizes to **13** under formation of the isocoumarin skeleton. The dihydropyrrole-fused isocoumarin intermediate **14** is formed through the intramolecular nucleophilic attack of the secondary amine group to the carbonyl carbon of **13**. Finally, intermediate **14** loses water to furnish pyrrole-fused isocoumarins **5** or **8**. It is worth mentioning that in the previous method instead of the formation of epoxy intermediate like **11** a transannular rearrangement was proposed for the product formation [38], because the formation of epoxy intermediate is less probable in the presence of a strong acid and a nucleophilic solvent as well as at higher temperatures.

**Figure 2:**
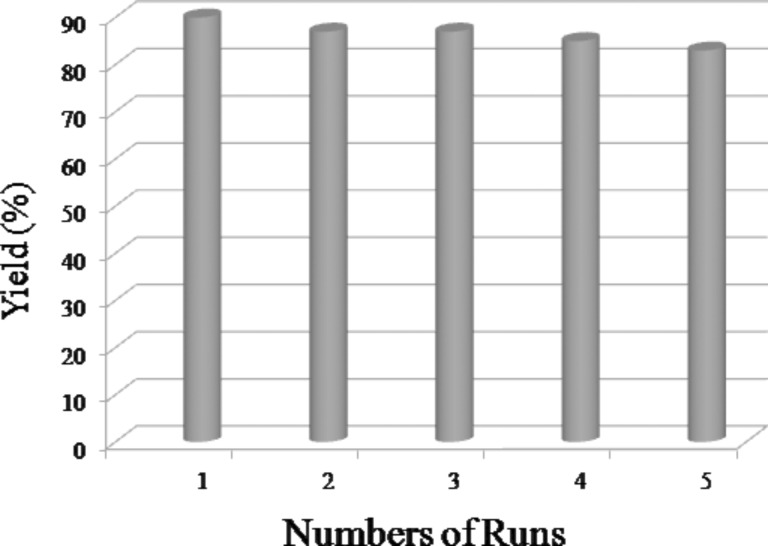
Reusability of SSA for the synthesis of pyrrole-fused isocoumarins.

Furthermore, a test with respect to recovery and reusability of SSA for the formation of **8a** was carried out. After heating the mixture of aniline and adduct **7** on the solid surface of SSA for 1 h, the product **8a** was isolated easily with ethylacetate by sonication of the reaction mixture. The recovered solid-supported SSA was reused five times, and the yield of the product **8a** varied from 90–83%, which indicates a substantial retention of catalytic activity and efficiency of SSA even after repeated application (Figure 2).

## Conclusion

In conclusion, a facile and convenient methodology has been developed for the synthesis of a diverse range of *N*-substituted pyrrole-fused isocoumarins in presence of the solid-supported Brønsted acid catalyst silica sulfuric acid (SSA). The methodology has a series of intrinsic advantages such as easy preparation of the solid supported SSA from chlorosulfonic acid and silica gel, less energy and manpower usage, easy product isolation/purification and operational simplicity, which lead to the synthetic route ‘‘benign by design’’. This is the first report, in which a rearrangement reaction has been carried out on the solid surface of SSA. Overall this greener and environmentally friendly method may attract the fellow chemists in chemical and pharmaceutical industries for the synthesis of biologically important pyrrole-fused isocoumarins.

## Experimental

**General information:** Starting materials and solvents were purchased from commercial suppliers and used without further purification. Melting points were determined in open capillary tubes and were uncorrected. IR spectra were recorded on a Perkin-Elmer 782 spectrophotometer. ^1^H (300 MHz) and ^13^C NMR (75 MHz) spectra were recorded on a Bruker 300 MHz instrument in CDCl_3_ and *d*_6_-DMSO. Elemental analyses (C, H and N) were performed by using a Perkin-Elmer 240C elemental analyzer. The X-ray diffraction data for crystallized compounds were collected with Mo Kα radiation at 296 K using the Bruker APEX-II CCD System. The crystals were positioned at 50 mm from the CCD. Frames were measured with a counting time of 5 s. Data analyses were carried out with the Bruker APEX2 and Bruker SAINT program. The structures were solved using direct methods with the SHELXS97 program.

**General experimental procedure for synthesis of pyrrole-fused isocoumarins 5 and 8:** A mixture of primary amines **2** (1.5 mmol) and dihydroxy indenofurans **6** or **7** (1.0 mmol) in chloroform (5 mL) was soaked in SSA (400 mg) by stirring for 10 min and then the solvent was removed under reduced pressure to get a solid mass. The solid mass was heated at 65 °C for 1 h under continuous stirring, until the complete disappearance of dihydroxyindenofuran was observed (as monitored by TLC). After cooling the solid mass to room temperature, ethylacetate (15 mL) was added to it, shaken thoroughly, ultra-sonicated and filtered to remove the SSA catalyst. The separated organic phase was evaporated under reduced pressure to get the crude product **5** or **8** which was purified by column chromatography (hexane/EtOAc).

**Reusability of the SSA:** After completion of the reaction, the product was isolated by ultra-sonication with ethylacetate and decanted. Then the separated solid catalyst SSA was dried under vacuum and reused directly for a new reaction set. The yield of the product **8a** varied from 90–83% after five consecutive cycles without any serious loss of efficiency of the solid supported SSA (Figure 2).

## Supporting Information

Supporting Information features detailed analytical data of the prepared compounds and a collection of NMR spectra.

File 1 Detailed analytical data.
