# Supplementary material for: Silica sulfuric acid: a reusable solid catalyst for one pot synthesis of densely substituted pyrrole-fused isocoumarins under solvent-free conditions
Source: Beilstein J Org Chem. 2013 Nov 4;9:2344–53. doi: 10.3762/bjoc.9.269 (PMC3869253; doi:10.3762/bjoc.9.269)
Supplement: File 1 — Detailed analytical data. [file Beilstein_J_Org_Chem-09-2344-s001.pdf]

Supporting Information

for

Silica sulfuric acid: a reusable solid catalyst for one pot synthesis of densely substituted pyrrole-fused isocoumarins under solvent-free conditions

Sudipta Pathak, Kamalesh Debnath and Animesh Pramanik*

Address: Department of Chemistry, University of Calcutta, 92, A. P. C. Road, Kolkata-700 009, India; Fax: +91-33-2351-9755; Tel: +91-33-2484-1647.

Email: Animesh Pramanik* - animesh_in2001@yahoo.co.in

* Corresponding author

Detailed analytical data

Contents **Page**

1. Preparation of silica sulfuric acid (SSA)……………..….………………...……S2
2. TGA analysis of SSA………………………………………….........................S2-S3
3. IR spectrum analysis………………………………...…………………...……S4-S5
4. NMR, IR, elemental analysis data……………………………………….…...S5-S10
5. ^{1}H NMR and ^{13}C NMR spectra of compounds **8**………………………………..S11-S25

Preparation of silica sulfuric acid:

15.0 g of silica gel (60–120 mesh) was washed with 1 mol/L HCl, followed by deionized water, 30% H_2O_2, and then again with deionized water. After thorough washing, the silica was dried overnight at 373 K in vacuum to get preconditioned silica gel. A 100-mL round-bottomed flask was charged with silica gel and fitted with a magnetic stirrer. Then chlorosulfonic acid (5.83 g, 50.0 mmol) was added dropwise to the silica gel at r.t. for a period of 0.5 h. Immediately HCl gas started to emerge from the reaction vessel. After addition of chlorosulfonic acid, the resulting mixture was stirred until there was no more HCl outgassing from the vessel. A white solid (silica sulfuric acid) of 19.0 g was obtained [1].

Thermal gravimetric analysis (TGA) of silica sulfuric acid (SSA):

The weight change of catalyst precursor was measured by using a TGA thermal analyzer apparatus under a flow of dry air. The temperature was raised from room temperature to 700 °C by using a linear programmer at a heating rate of 10 °C/min. The sample weight was 18.7 mg. The TGA curve for the catalyst is illustrated in Figure S1. The weight losses found from TGA measurements agree fairly well with those expected for the decomposition of silica sulfuric acid to silica. For the catalyst, the thermo gravimetric curve seems to indicate a decomposition that is considered to be due to the removal of silica sulfuric acid (130–360 °C). It goes along with a total overall weight loss 29.29%, which solely represents the decomposition of the silica sulfuric acid to silica.[2]

References

1. Zolfigol, M. A. *Tetrahedron* **2001,** *57,* 9509.

2. Shaterian, H. R.; Ghashang, M.; Feyzi, M. *Applied Catalysis A: General* **2008,** *345,* 128.

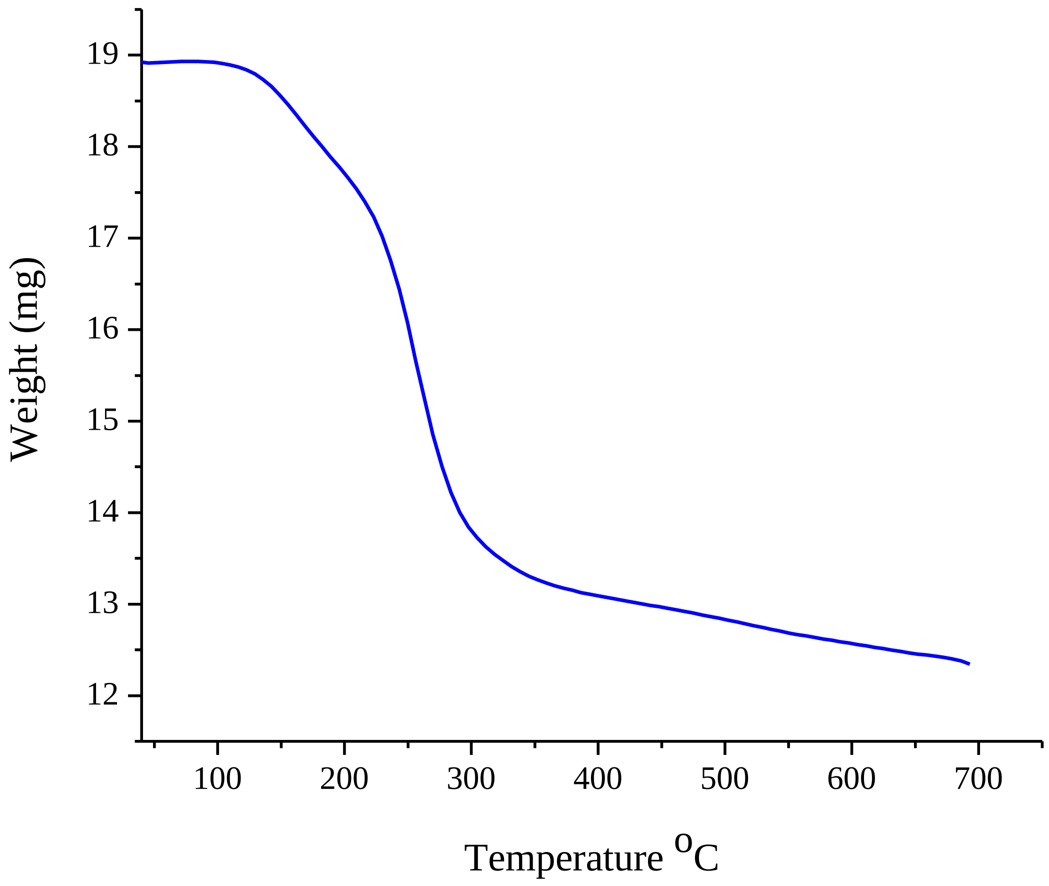

Figure S1 TGA curve of the silica sulfuric acid.

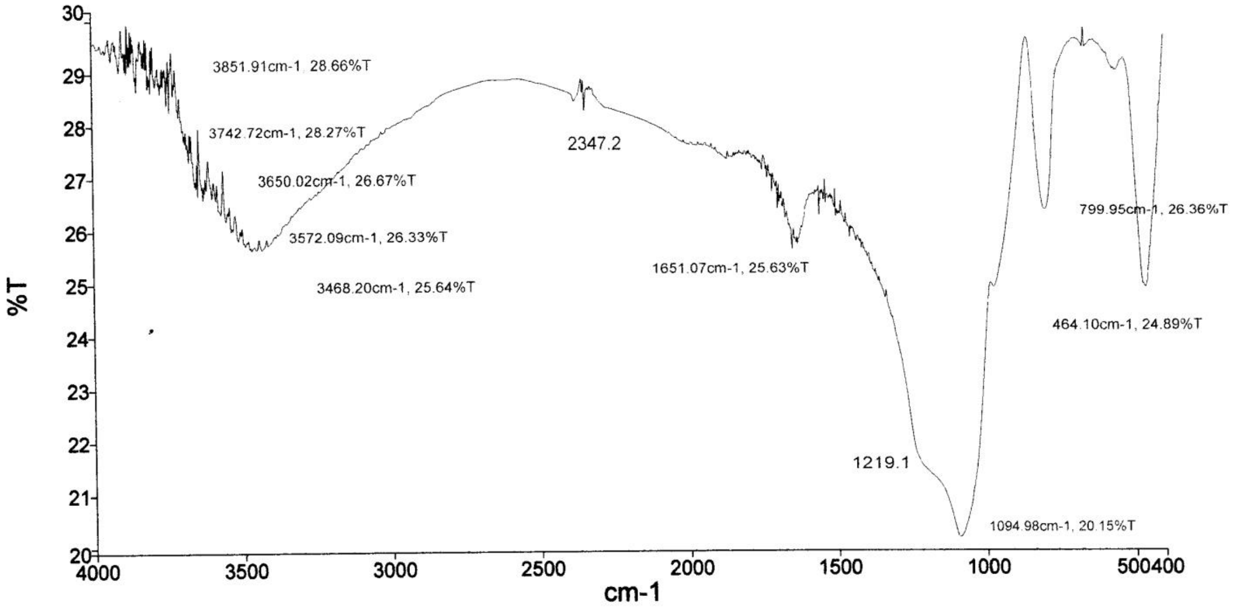

Figure S2: IR spectrum of Silica.

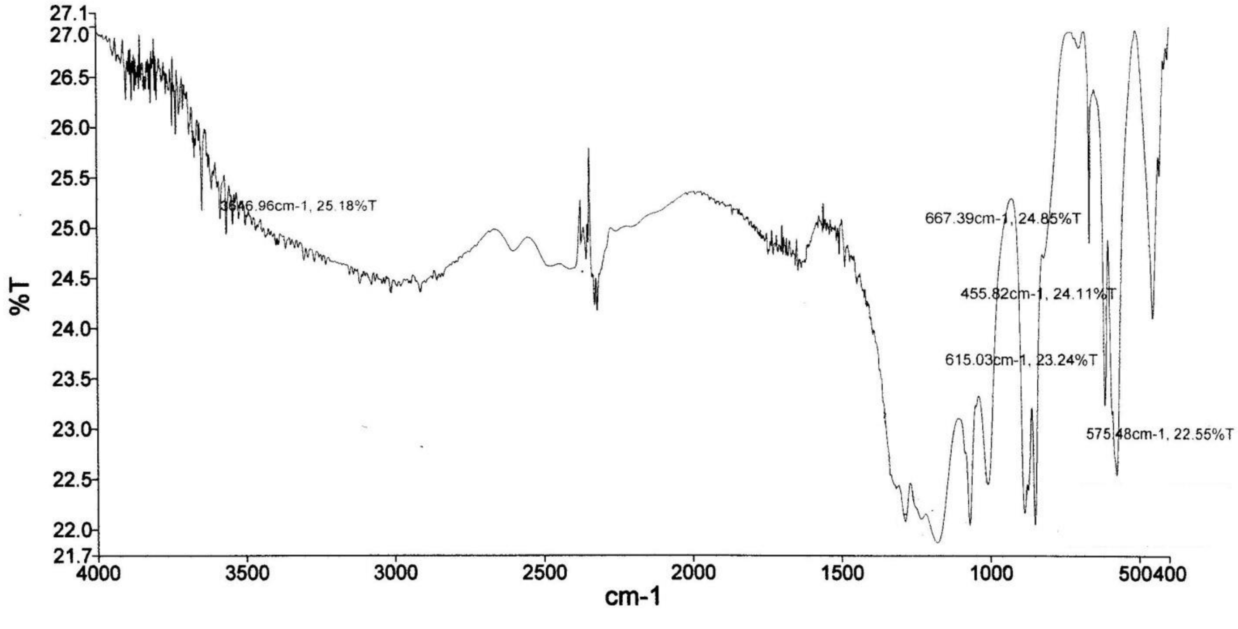

Figure S3: IR spectrum of silica sulfuric acid.

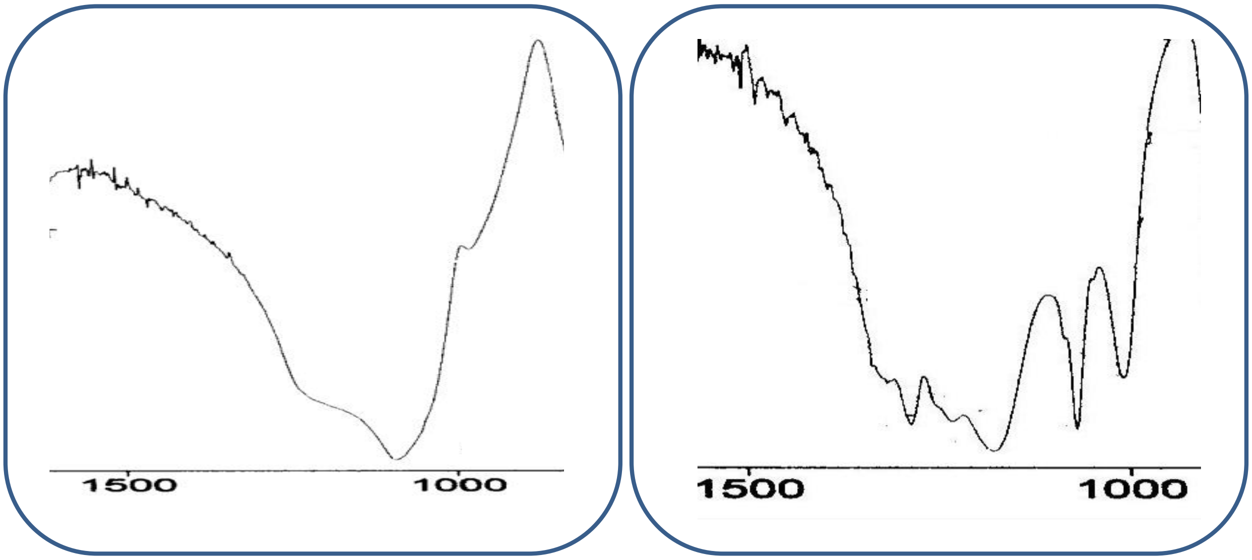

(a) IR spectrum of silica

(b) IR spectrum of silica sulfuric acid

Figure S4: Comparison of IR spectrums of silica gel and silica sulfuric aicd

Analysis of IR spectrum: The FT-IR spectrums of the catalyst were shown in Figure S2, S3 and S4. The catalyst is solid and solid state IR spectrum was recorded using the KBr disk technique. For silica (SiO_2), the major peaks are broad band Si–O–Si stretching from 1100 to 1000 cm^{-1}. For sulfonic acid functional group, the FT-IR absorption range of the O=S=O peaks lies in 1180–1250 cm^{-1}.

Spectral data of the compounds

2-Methyl-5-oxo-1-phenyl-1,5-dihydro-4-oxa-1-azacyclopenta[*a*]naphthalene-3-carboxylic acidethylester (8a). Yellow solid; mp 208-210 ºC; ^{1}H NMR ($CDCl_3$, 300MHz) δ 8.29 (d, *J* = 7.8 Hz, 1H), 7.65-7.62 (m, 3H), 7.42-7.39 (m, 1H), 7.32-7.30 (m, 1H), 7.27-7.20 (m, 2H), 6.36 (d, *J* = 7.8 Hz, 1H), 4.39 (q, *J* = 6.9 Hz, 2H), 2.38 (s, 3H), 1.43 (t, *J* = 7.2 Hz, 3H); ^{13}C NMR ($CDCl_3$, 75 MHz) δ 163.6, 162.1, 139.7, 137.3, 134.2, 131.7, 130.3, 130.2, 130.2, 130.0, 128.5, 125.4,

118.4, 118.0, 112.9, 102.1, 60.1, 14.5, 12.0; IR(KBr): 1722 cm^{-1}; Anal calcd for $C_{21}H_{17}NO_4$: C, 72.61; H, 4.93; N, 4.03 % Found C, 72.50; H, 4.87; N, 3.98%.

1-(4-Fluorophenyl)-2-methyl-5-oxo-1,5-dihydro-4-oxa-1-azacyclopenta[*a*]naphthalene-3-carboxylicacidethylester (**8b**). Yellow solid; mp 252-254 ºC; ^{1}H NMR ($CDCl_3$, 300MHz) 8.23 (d, J = 7.8 Hz, 1H), 7.50-7.46 (m, 2H), 7.37-7.31 (m, 3H), 7.21 (t, J = 8.1 Hz, 1H), 6.38 (d, J = 8.1 Hz, 1H), 4.32 (q, J = 6.9 Hz, 2H), 2.36 (s, 3H), 1.41 (t, J = 7.2 Hz, 3H); ^{13}C NMR ($CDCl_3$, 75 MHz, F coupled ^{13}C spectra) δ 164.8, 163.3, 161.9, 161.5, 140.7, 139.8, 134.3, 133.2, 131.7, 130.6, 130.5, 130.1, 125.5, 118.2, 118.2, 117.5, 117.1, 112.9, 102.2, 60.1, 14.4, 11.9; IR(KBr): 1725 cm^{-1}; Anal calcd for $C_{21}H_{16}FNO_4$: C, 69.04; H, 4.41; N, 3.83 % Found 68.96; H, 4.36; N, 3.80 %.

1-(2-Chlorophenyl)-2-methyl-5-oxo-1,5-dihydro-4-oxa-1-azacyclopenta[*a*]naphthalene-3-carboxylicacidethylester (**8c**). Yellow solid; mp 233-235 ºC; ^{1}H NMR ($CDCl_3$, 300MHz) δ 8.32 (d, J = 7.8 Hz, 1H), 7.69-7.55 (m, 4H), 7.35-7.26 (m, 2H), 6.31 (d, J = 7.8 Hz, 1H), 4.42 (q, J = 7.2 Hz, 2H), 2.37 (s, 3H), 1.45 (t, J = 7.2 Hz, 3H); ^{13}C NMR ($CDCl_3$, 75 MHz) δ 163.5, 162.0, 140.9, 139.5, 135.1, 134.6, 133.9, 131.8, 131.0, 130.7, 130.6, 130.2, 128.5, 125.6, 118.1, 117.5, 112.4, 102.7, 60.2, 14.5, 11.6; IR(KBr): 1724 cm^{-1}; Anal calcd for $C_{21}H_{16}ClNO_4$: C, 66.06; H, 4.22; N, 3.67 % Found C, 66.17; H, 4.15; N, 3.61 %.

1-(3-Chlorophenyl)-2-methyl-5-oxo-1,5-dihydro-4-oxa-1-azacyclopenta[*a*]naphthalene-3-carboxylicacidethylester (**8d**). Yellow solid; mp 230-232 ºC; ^{1}H NMR ($CDCl_3$, 300MHz) δ 8.20 (d, J = 7.8 Hz, 1H), 7.59-7.49 (m, 2H), 7.39 (d, J = 7.8 Hz, 1H), 7.32-7.25 (m, 2H), 7.20-7.15 (m, 1H), 6.33 (d, J = 8.1 Hz, 1H), 4.28 (q, J = 6.9 Hz, 2H), 2.31 (s, 3H), 1.34 (t, J = 6.9 Hz, 3H); ^{13}C NMR ($CDCl_3$, 75 MHz) δ 163.3, 161.8, 140.9, 139.6, 138.4, 135.9, 134.4, 131.8, 131.2,

130.4, 130.0, 128.9, 127.0, 125.7, 118.2, 118.1, 112.8, 102.5, 60.1, 14.4, 12.0; IR(KBr): 1721 cm^{-1}; Anal calcd for $C_{21}H_{16}ClNO_4$: C, 66.06; H, 4.22; N, 3.67 % Found C, 66.16; H, 4.18; N, 3.63 %.

1-(4-Chlorophenyl)-2-methyl-5-oxo-1,5-dihydro-4-oxa-1-azacyclopenta[*a*]naphthalene-3-carboxylicacidethylester (**8e**). Yellow solid; mp 218-220 ºC; ^{1}H NMR ($CDCl_3$, 300MHz) δ 8.23 (d, *J* = 7.8 Hz, 1H), 7.63 (d, *J* = 6.9 Hz, 2H), 7.43 (d, *J* = 8.4 Hz, 2H), 7.32 (t, *J* = 7.2 Hz, 1H), 7.24 (t, *J* = 7.2 Hz, 1H), 6.42 (d, *J* = 8.1 Hz, 1H), 4.32 (q, *J* = 6.9 Hz, 2H), 2.36 (s, 3H), 1.40 (t, *J* = 7.2 Hz, 3H); ^{13}C NMR ($CDCl_3$, 75 MHz) δ 163.2, 161.8, 140.8, 139.6, 136.1, 135.8, 134.3, 131.7, 131.7, 130.5, 130.0, 125.5, 118.2, 117.9, 112.7, 102.3, 60.1, 14.4, 11.9; IR(KBr): 1719 cm^{-1}; Anal calcd for $C_{21}H_{16}ClNO_4$: C, 66.06; H, 4.22; N, 3.67 % Found C, 65.93; H, 4.15; N, 3.60 %.

1-(4-Methoxyphenyl)-2-methyl-5-oxo-1,5-dihydro-4-oxa-1-azacyclopenta[*a*]naphthalene-3-carboxylicacidethylester (**8f**). Yellow solid; mp 194-196 ºC; ^{1}H NMR ($CDCl_3$, 300MHz) δ 8.23 (d, *J* = 8.1 Hz, 1H), 7.28-7.14 (m, 4H), 7.03 (d, *J* = 8.7 Hz, 2H), 6.39 (d, *J* = 8.1 Hz, 1H), 4.32 (q, *J* = 6.9 Hz, 2H), 3.87 (s, 3H), 2.30 (s, 3H), 1.35 (t, *J* = 7.2 Hz, 3H); ^{13}C NMR ($CDCl_3$, 75 MHz) δ 163.7, 161.9, 160.5, 140.7, 140.1, 134.3, 131.7, 130.5, 129.7, 129.5, 125.4, 118.4, 118.0, 115.3, 113.0, 101.9, 60.1, 55.6, 14.5, 11.9; IR(KBr): 1716 cm^{-1}; Anal calcd for $C_{22}H_{19}NO_5$: C, 70.02; H, 5.07; N, 3.71 % Found C, 70.14; H, 5.00; N, 3.66 %.

1-(3-Methoxyphenyl)-2-methyl-5-oxo-1,5-dihydro-4-oxa-1-azacyclopenta[*a*]naphthalene-3-carboxylicacidethylester (**8g**). Yellow solid; mp 198-200 ºC; ^{1}H NMR ($CDCl_3$, 300MHz) δ 8.26 (d, *J* = 7.8 Hz, 1H), 7.52 (t, *J* = 8.1 Hz, 1H), 7.33 (t, *J* = 7.8 Hz, 1H), 7.27-7.15 (m, 2H), 6.99-6.95 (m, 2H), 6.46 (d, *J* = 8.1 Hz, 1H), 4.36 (q, *J* = 6.9 Hz, 2H), 3.88 (s, 3H), 2.39 (s, 3H),

1.41 (t, *J* = 6.9 Hz, 3H); ^{13}C NMR ($CDCl_3$, 75 MHz) δ163.5, 162.1, 160.9, 140.7, 139.6, 138.2, 134.3, 131.6, 130.9, 130.3, 125.4, 120.5, 118.5, 117.9, 115.9, 114.0, 112.8, 102.0, 60.1, 55.6, 14.5, 11.9; IR(KBr): 1720 cm^{-1}; Anal calcd for $C_{22}H_{19}NO_5$: C, 70.02; H, 5.07; N, 3.71 % Found C, 70.15; H, 5.02; N, 3.67 %.

1-(3-Hydroxyphenyl)-2-methyl-5-oxo-1,5-dihydro-4-oxa-1-azacyclopenta[*a*]naphthalene-3-carboxylicacidethylester (**8h**). Yellow solid; mp 254-256 ºC; ^{1}H NMR (d_6-DMSO, 300MHz) δ 10.13 (bs, 1H), 8.13 (d, *J* = 7.8 Hz, 1H), 7.53-7.43 (m, 2H), 7.32 (t, *J* = 7.5 Hz, 1H), 7.08 (d, *J* = 8.1 Hz, 1H), 6.95 (d, *J* = 7.5 Hz, 1H), 6.87 (s, 1H), 6.44 (d, *J* = 8.1 Hz, 1H), 4.24 (q, *J* = 6.9 Hz, 2H), 2.29 (s, 3H), 1.29 (t, *J* = 6.6 Hz, 3H); ^{13}C NMR (d_6-DMSO, 75 MHz) δ 162.8, 161.5, 159.1, 140.8, 139.5, 137.8, 135.2, 131.5, 131.4, 130.2, 126.2, 119.1, 118.7, 117.7, 117.5, 115.5, 112.5, 101.2, 59.8, 14.7, 12.1; IR(KBr): 3350, 1722 cm^{-1}; Anal calcd for $C_{21}H_{17}NO_5$: C, 69.41; H, 4.72; N, 3.85 % Found C, 69.29; H, 4.67; N, 3.79 %.

2-Methyl-5-oxo-1-*p*-tolyl-1,5-dihydro-4-oxa-1-azacyclopenta[*a*]naphthalene-3-carboxylic acidethylester (**8i**). Yellow solid; mp 202-204 ºC; ^{1}H NMR ($CDCl_3$, 300MHz) δ 8.23 (d, *J* = 8.1 Hz, 1H), 7.38 (d, *J* = 8.1 Hz, 2H), 7.30-7.15 (m, 4H), 6.39 (d, *J* = 8.1 Hz, 1H), 4.33 (q, *J* = 7.2 Hz, 2H), 2.49 (s, 3H), 2.33 (s, 3H), 1.38 (t, *J* = 7.2 Hz, 3H); ^{13}C NMR ($CDCl_3$, 75 MHz) δ 163.6, 162.1, 140.7, 140.2, 139.8, 134.6, 134.2, 131.6, 130.8, 130.4, 128.2, 125.3, 118.5, 118.0, 112.8, 101.9, 60.0, 21.3, 14.5, 11.9; IR(KBr): 1720 cm^{-1}; Anal calcd for $C_{22}H_{19}NO_4$: C, 73.12; H, 5.30; N, 3.88 % Found C, 73.01; H, 5.26; N, 3.82 %.

1-(2,4-Dimethylphenyl)-2-methyl-5-oxo-1,5-dihydro-4-oxa-1-azacyclopenta[*a*]naphthalene-3-carboxylicacidethylester (**8j**). Yellow solid; mp 190-192 ºC; ^{1}H NMR ($CDCl_3$, 300MHz) δ 8.27 (d, *J* = 7.8 Hz, 1H), 7.29-7.11 (m, 5H), 6.31 (d, *J* = 7.8 Hz, 1H), 4.35 (q, *J* = 6.9, 2H), 2.44

(s, 3H), 2.29 (s, 3H), 1.86 (s, 3H), 1.38 (t, *J* = 7.2 Hz, 3H); ^{13}C NMR ($CDCl_3$, 75 MHz) δ 163.7, 162.2, 140.7, 140.5, 139.2, 136.4, 134.6, 133.5, 132.3, 131.7, 130.5, 128.4, 128.2, 125.5, 117.9, 117.7, 112.2, 102.2, 60.1, 21.3, 17.0, 14.5, 11.6; IR(KBr): 1717 cm^{-1}; Anal calcd for $C_{23}H_{21}NO_4$: C, 73.58; H, 5.64; N, 3.73 % Found C, 73.71; H, 5.56; N, 3.69 %.

1-(4-Bromophenyl)-2-methyl-5-oxo-1,5-dihydro-4-oxa-1-azacyclopenta[*a*]naphthalene-3-carboxylicacidethylester (**8k**). Yellow solid; mp 212-214 ºC; ^{1}H NMR ($CDCl_3$, 300MHz) δ 8.27 (d, *J* = 7.5 Hz, 1H), 7.80 (d, *J* = 8.4 Hz, 2H), 7.38-7.22 (m, 4H), 6.43 (d, *J* = 8.1 Hz, 1H), 4.36 (q, *J* = 6.9 Hz, 2H), 2.36 (s, 3H), 1.41 (t, *J* = 7.2 Hz, 3H); ^{13}C NMR ($CDCl_3$, 75 MHz) δ 163.3, 161.8, 140.9, 139.6, 136.3, 134.3, 133.5, 131.8, 130.2, 130.0, 125.6, 124.2, 118.2, 118.0, 112.7, 102.5, 60.1, 14.4, 12.0; IR(KBr): 1721 cm^{-1}; Anal calcd for $C_{21}H_{16}BrNO_4$: C, 59.17; H, 3.78; N, 3.29 % Found 59.05; H, 3.70; N, 3.22 %.

2-Methyl-1-(4-nitrophenyl)-5-oxo-1,5-dihydro-4-oxa-1-azacyclopenta[*a*]naphthalene-3-carboxylicacidethylester (**8l**). Yellow solid; mp 260-262 ºC; ^{1}H NMR ($CDCl_3$, 300MHz) δ 8.52 (d, *J* = 8.1 Hz, 2H), 8.34 (d, *J* = 7.8, 1H), 7.64 (d, *J* = 8.7 Hz, 2H), 7.39-7.30 (m, 2H), 6.36 (d, *J* = 7.5 Hz, 1H), 4.41 (q, *J* = 7.2 Hz, 2H), 2.41 (s, 3H), 1.44 (t, *J* = 7.2 Hz, 3H); IR(KBr): 1726 cm^{-1}; Anal calcd for $C_{21}H_{16}N_2O_6$: C, 64.28; H, 4.11; N, 7.14 % Found C, 64.19; H, 4.05; N, 7.08 %.

1-Benzyl-2-methyl-5-oxo-1,5-dihydro-4-oxa-1-azacyclopenta[*a*]naphthalene-3-carboxylic acidethylester (**8m**). Yellow solid; mp 180-182 ºC; ^{1}H NMR ($CDCl_3$, 300MHz) δ 8.27 (d, *J* = 7.8 Hz, 1H), 7.44 (t, *J* = 8.4 Hz, 1H), 7.38-7.21 (m, 5H), 7.02 (d, *J* = 6.9 Hz, 2H), 5.54 (s, 2H), 4.38 (q, *J* = 7.2 Hz, 2H), 2.61 (s, 3H), 1.44 (t, *J* = 6.9 Hz, 3H); ^{13}C NMR ($CDCl_3$, 75 MHz) δ 163.5, 161.9, 140.9, 139.2, 135.3, 134.7, 131.8, 130.1, 129.3, 128.0, 125.4, 125.3, 118.6, 118.0,

111.9, 101.8, 60.1, 48.7, 14.5, 10.9; IR(KBr): 1720 cm^{-1}; Anal calcd for $C_{22}H_{19}NO_4$: C, 73.12; H, 5.30; N, 3.88 % Found C, 73.21; H, 5.25; N, 3.84 %.

2-Methyl-5-oxo-1-propyl-1,5-dihydro-4-oxa-1-azacyclopenta[*a*]naphthalene-3-carboxylic acidethylester (**8n**). Yellow solid; mp 132-134 ºC; ^{1}H NMR ($CDCl_3$, 300MHz) δ 8.28 (d, *J* = 7.8 Hz, 1H), 7.64 (t, *J* = 7.8 Hz, 1H), 7.54 (d, *J* = 8.1 Hz, 1H), 7.29 (t, *J* = 7.2 Hz, 1H), 4.35 (q, *J* = 6.9 Hz, 2H), 4.17 (t, *J* = 7.2 Hz, 2H), 2.61 (s, 3H), 1.86-1.79 (m, 2H), 1.42 (t, *J* = 6.9 Hz, 3H), 1.03 (t, *J* = 7.2 Hz, 3H); ^{13}C NMR ($CDCl_3$, 75 MHz) δ 163.5, 161.9, 140.9, 138.5, 134.8, 131.9, 130.3, 125.1, 118.3, 117.8, 110.9, 101.0, 59.8, 46.7, 23.3, 14.5, 11.0, 10.9; IR(KBr): 1720 cm^{-1}; Anal calcd for $C_{18}H_{19}NO_4$: C, 68.99; H, 6.11; N, 4.47 % Found C, 68.88; H, 6.04; N, 4.41 %.

1-Butyl-2-methyl-5-oxo-1,5-dihydro-4-oxa-1-azacyclopenta[*a*]naphthalene-3-carboxylic acidethylester (**8o**). Yellow solid; mp 125-127 ºC; ^{1}H NMR ($CDCl_3$, 300MHz) δ 8.29 (d, *J* = 7.8 Hz, 1H), 7.65 (t, *J* = 7.5 Hz, 1H), 7.56 (d, *J* = 8.1 Hz, 1H), 7.30 (t, *J* = 7.8 Hz, 1H), 4.36 (q, *J* = 7.2 Hz, 2H), 4.20 (t, *J* = 7.8 Hz, 2H), 2.62 (s, 3H), 1.79-1.74 (m, 2H), 1.49-1.40 (m, 5H), 1.00 (t, *J* = 7.2 Hz, 3H); ^{13}C NMR ($CDCl_3$, 75 MHz) δ 163.5, 161.9, 141.0, 138.4, 134.7, 132.0, 130.4, 125.1, 118.3, 117.8, 110.9, 101.0, 59.8, 45.1, 32.1, 19.8, 14.5, 13.6, 11.0; IR(KBr): 1719 cm^{-1}; Anal calcd for $C_{19}H_{21}NO_4$: C, 69.71; H, 6.47; N, 4.28 % Found C, 69.61; H, 6.41; N, 4.20 %.

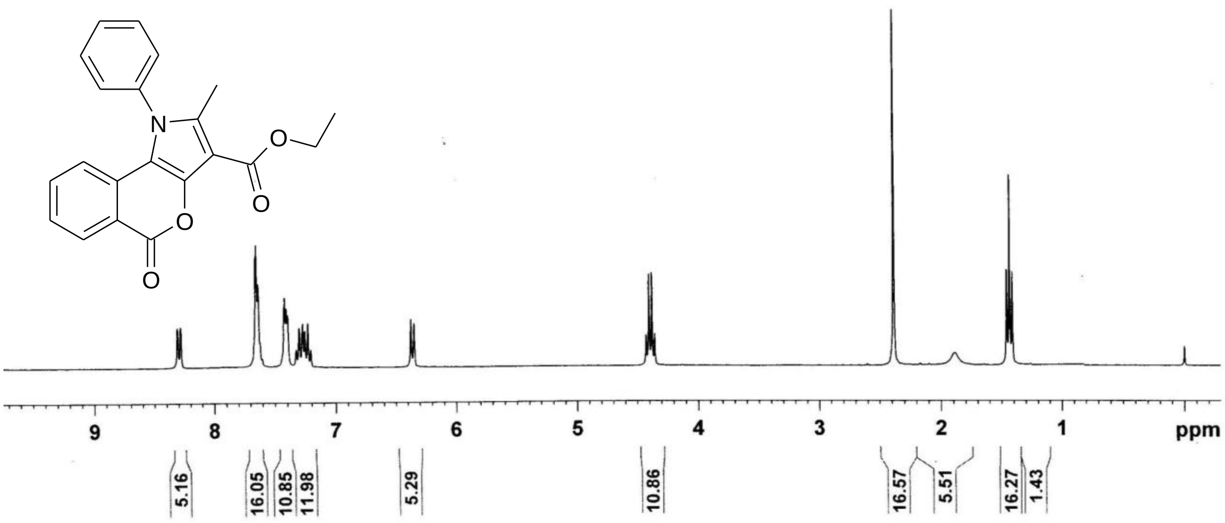

^{1}H NMR of Compound **8a** in $CDCl_3$

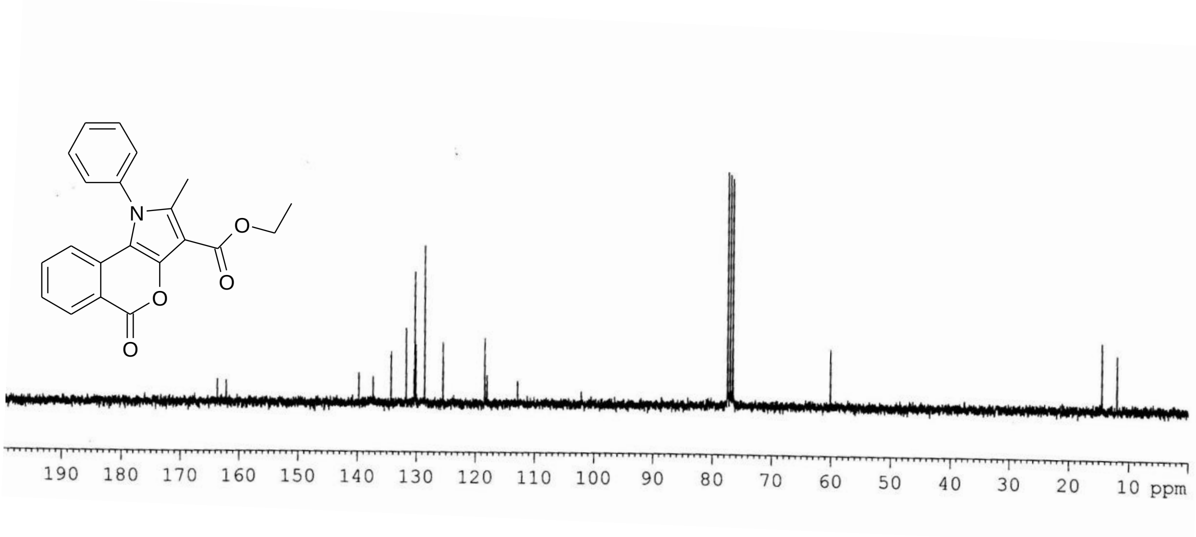

^{13}C NMR of Compound **8a** in $CDCl_3$

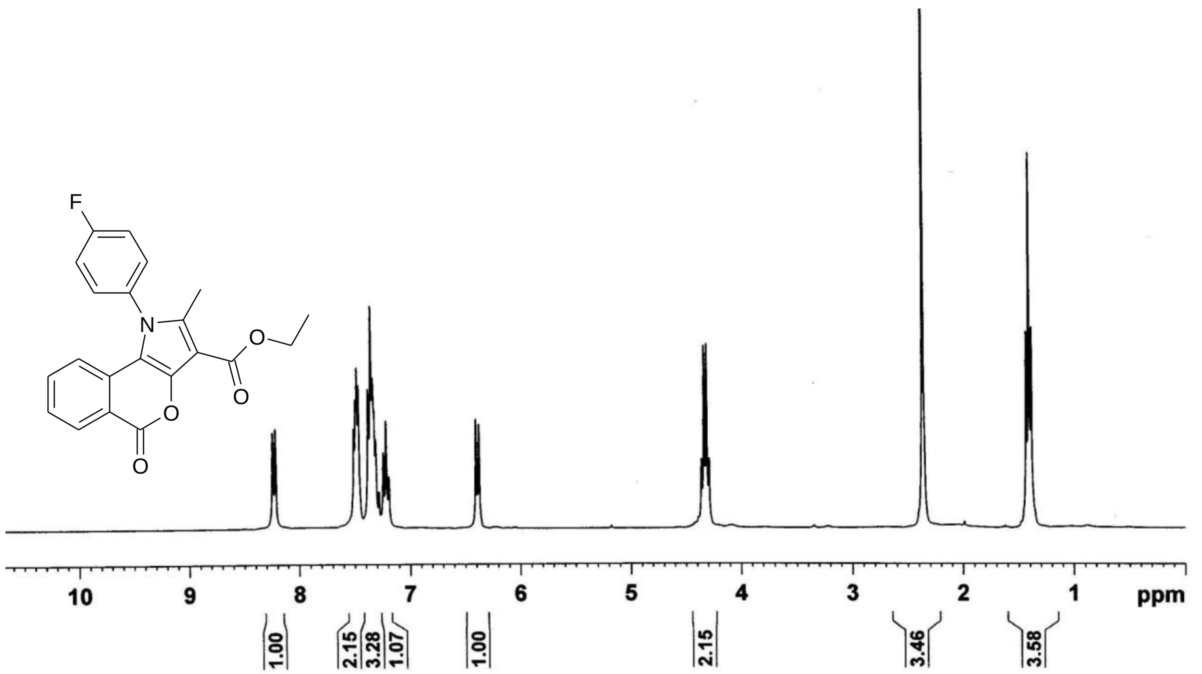

^{1}H NMR of Compound **8b** in $CDCl_3$

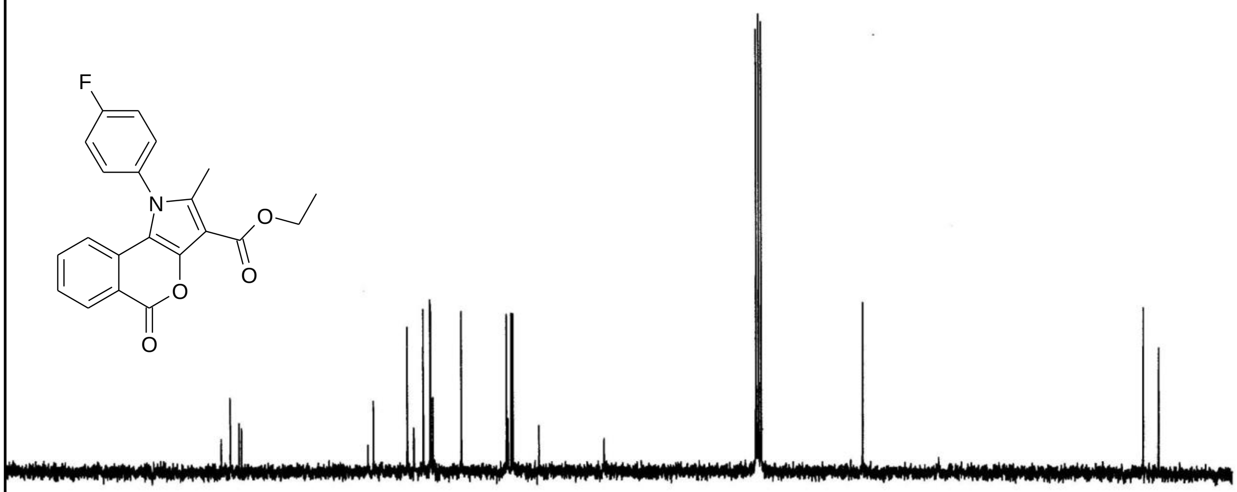

^{13}C NMR of Compound **8b** in $CDCl_3$

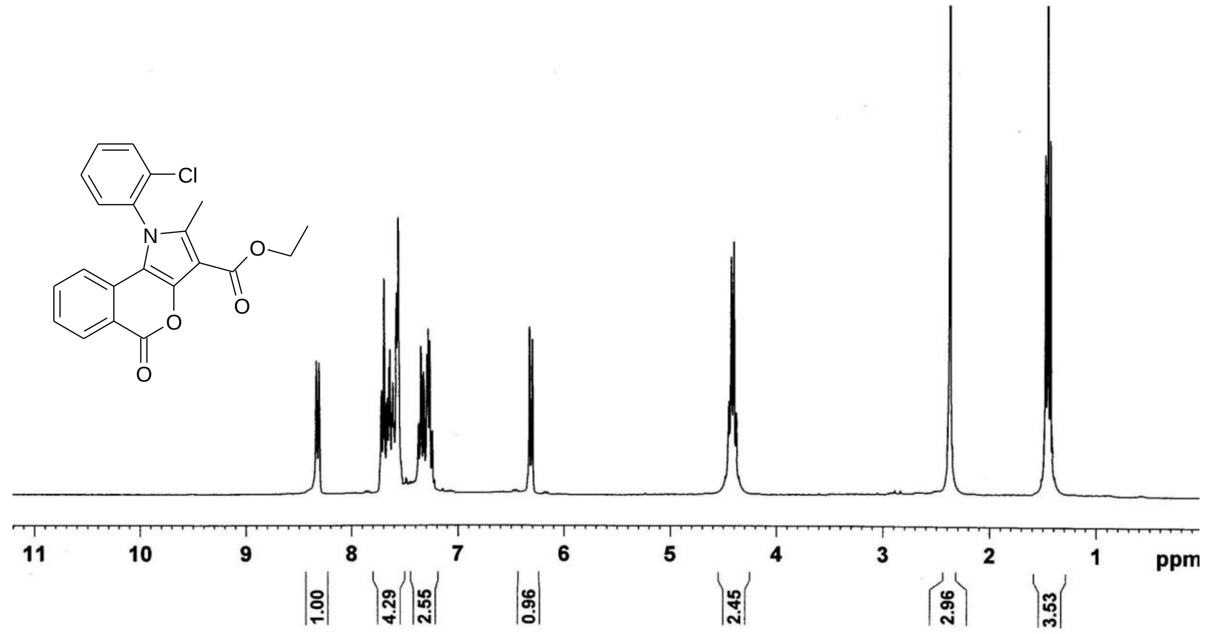

^{1}H NMR of Compound **8c** in $CDCl_3$

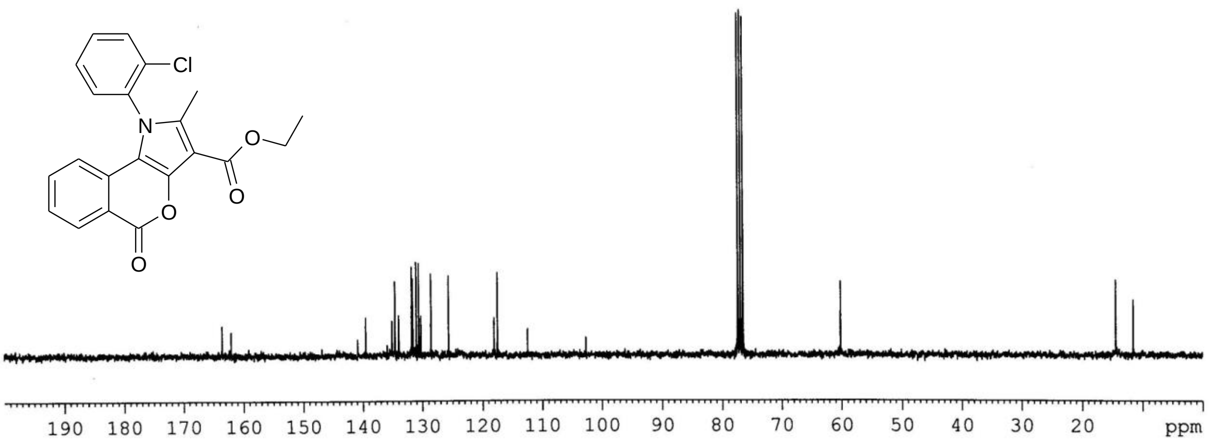

^{13}C NMR of Compound **8c** in $CDCl_3$

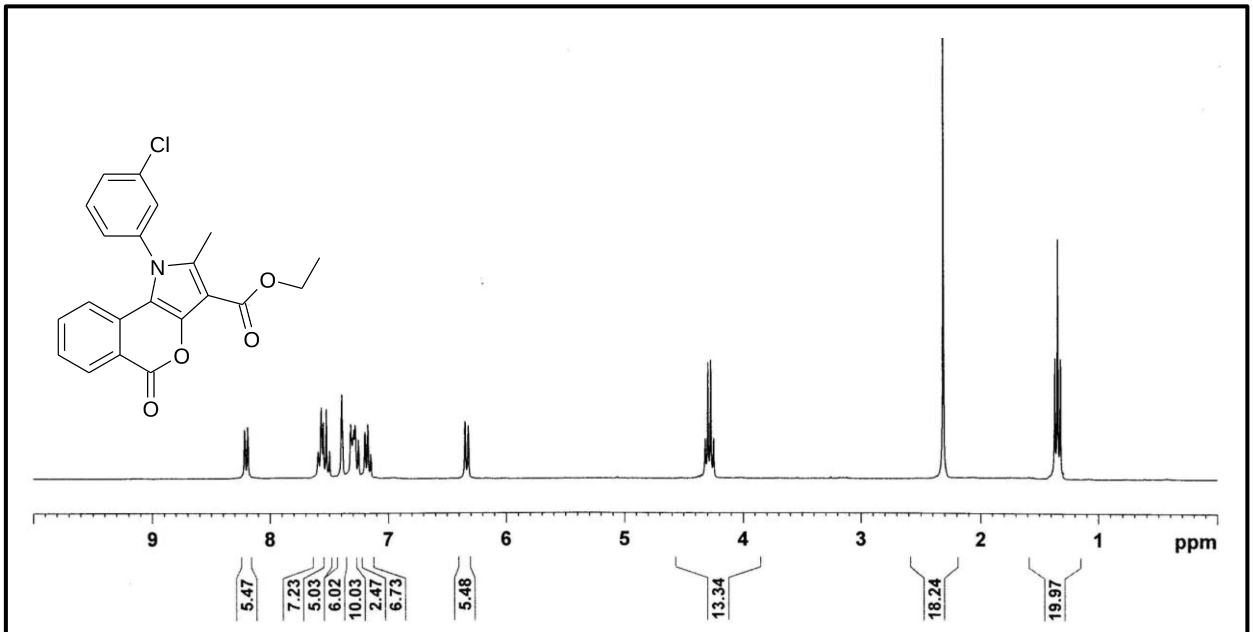

^{1}H NMR of Compound **8d** in $CDCl_3$

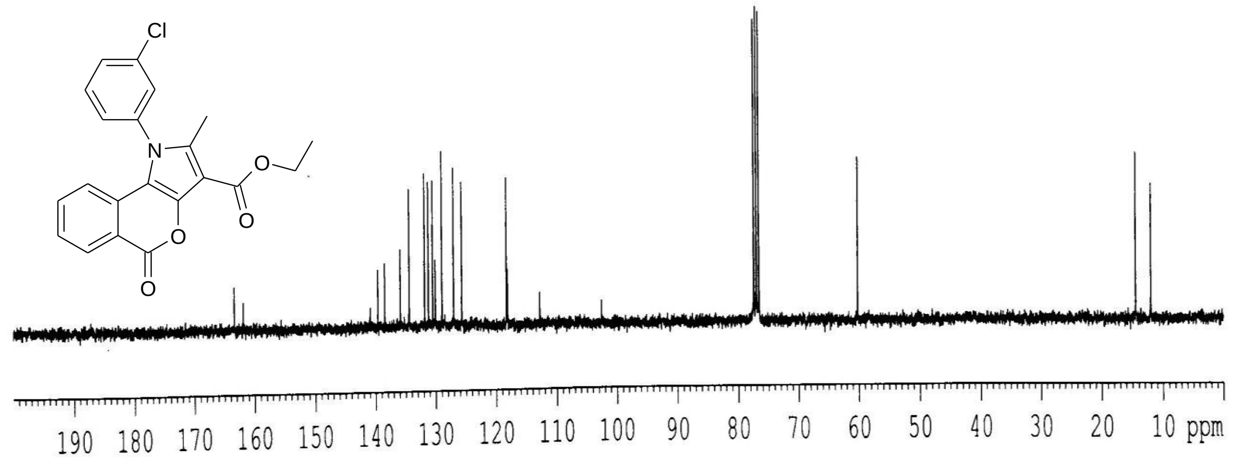

^{13}C NMR of Compound **8d** in $CDCl_3$

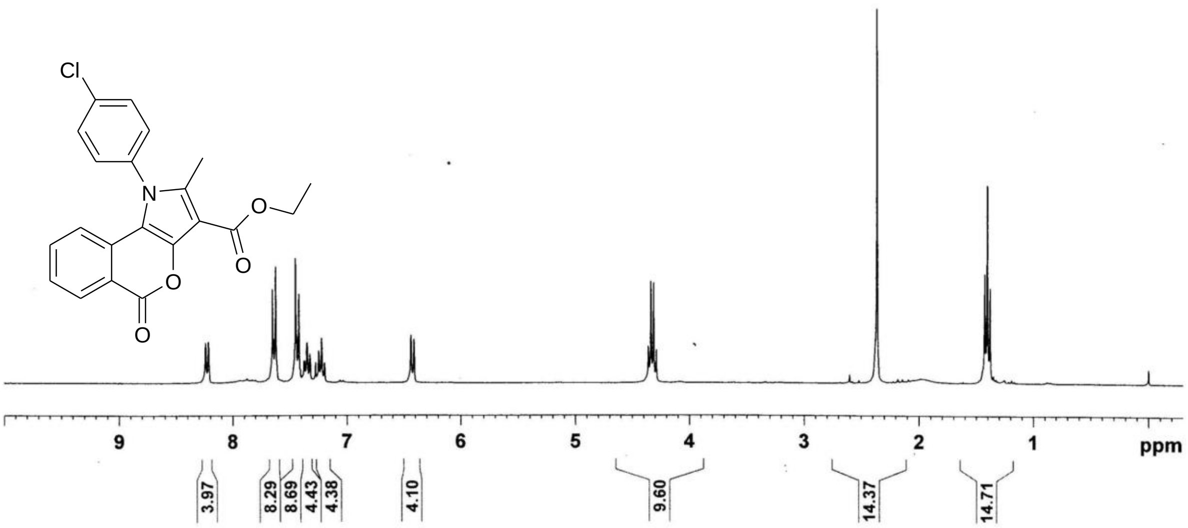

^{1}H NMR of Compound **8e** in $CDCl_3$

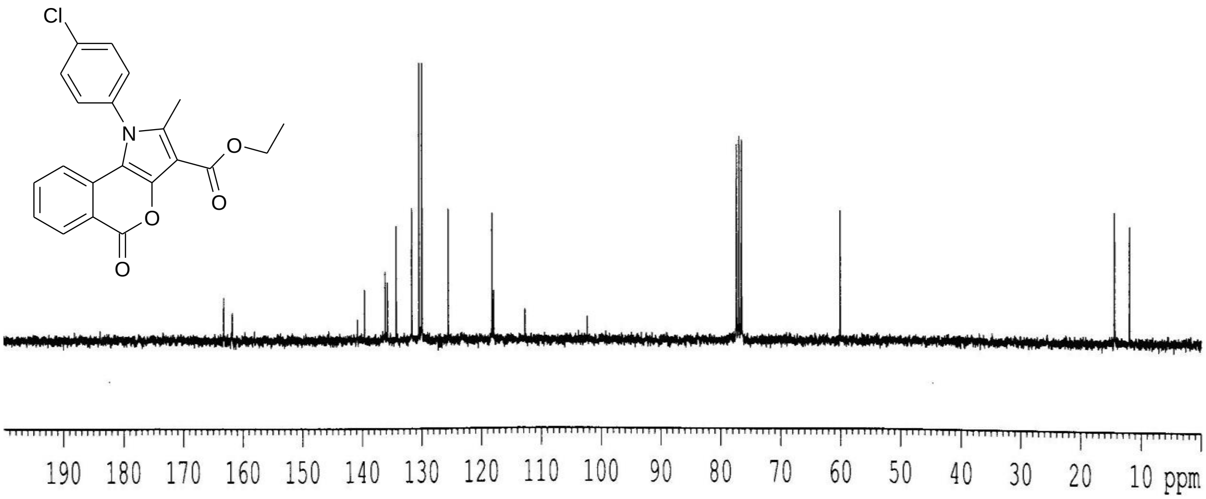

^{13}C NMR of Compound **8e** in $CDCl_3$

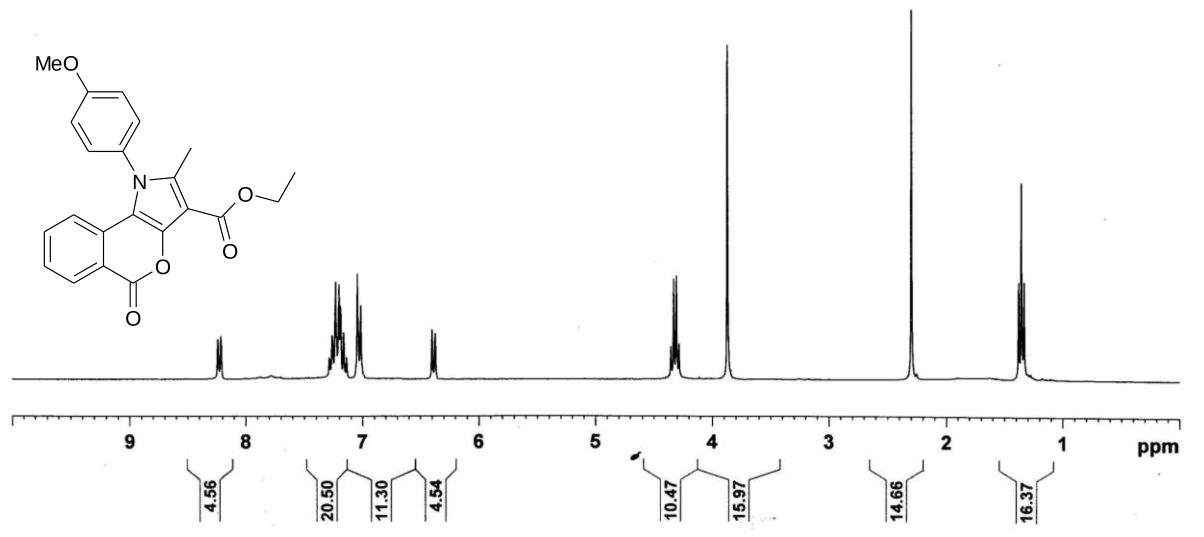

^{1}H NMR of Compound **8f** in $CDCl_3$

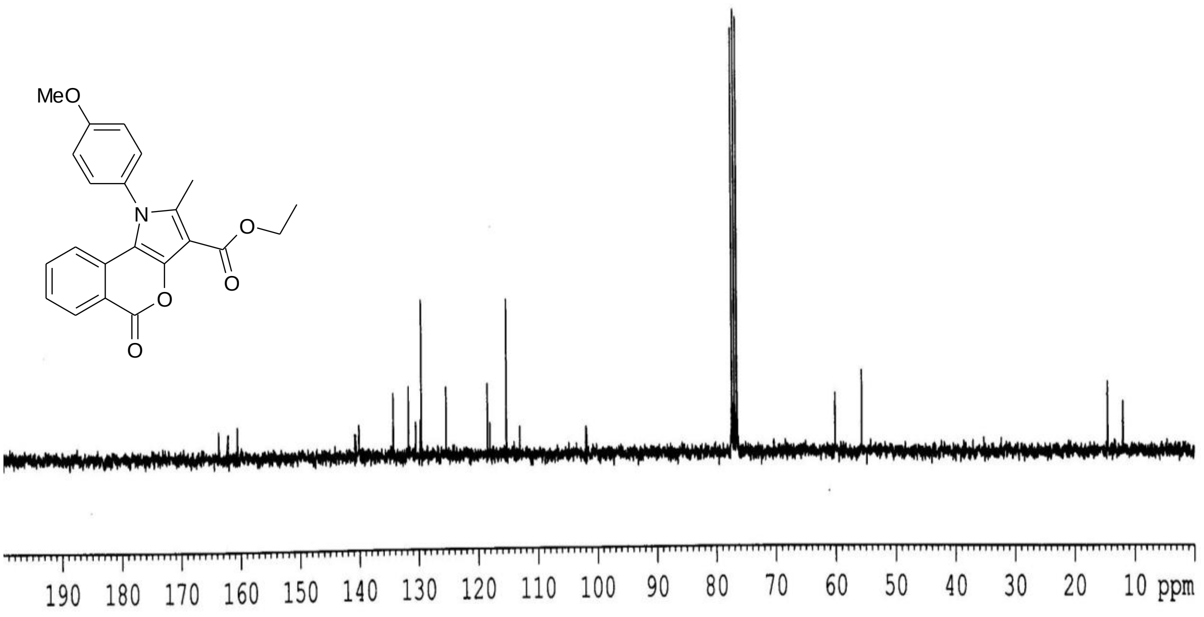

^{13}C NMR of Compound **8f** in $CDCl_3$

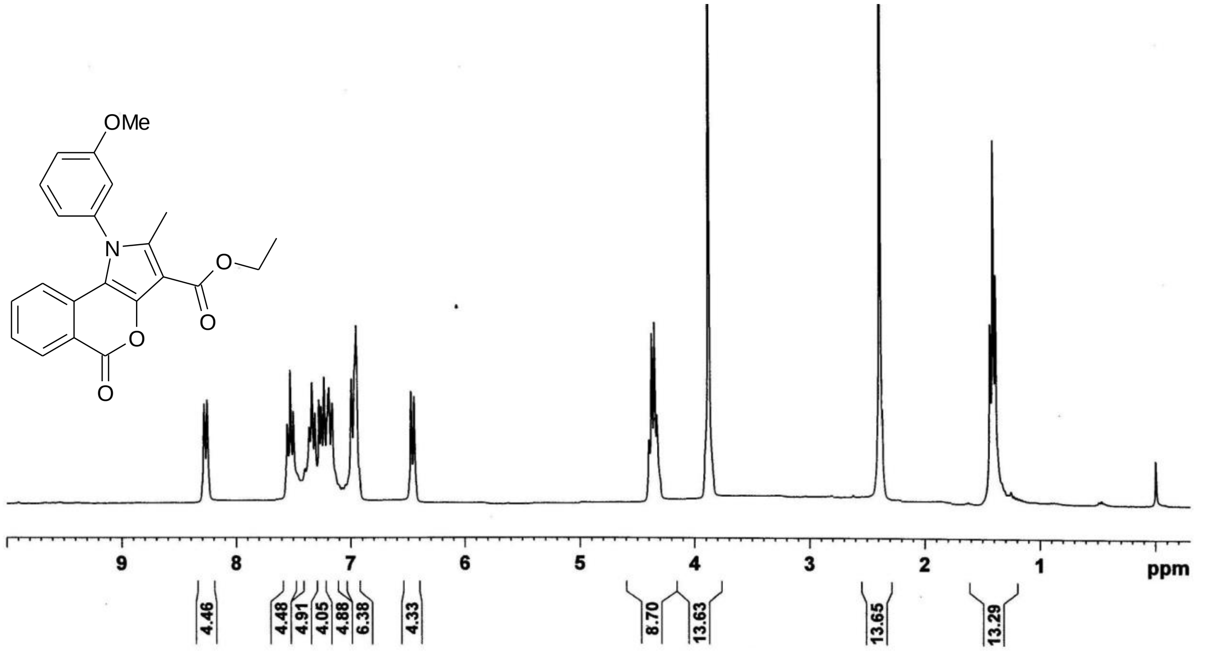

^{1}H NMR of Compound **8g** in $CDCl_3$

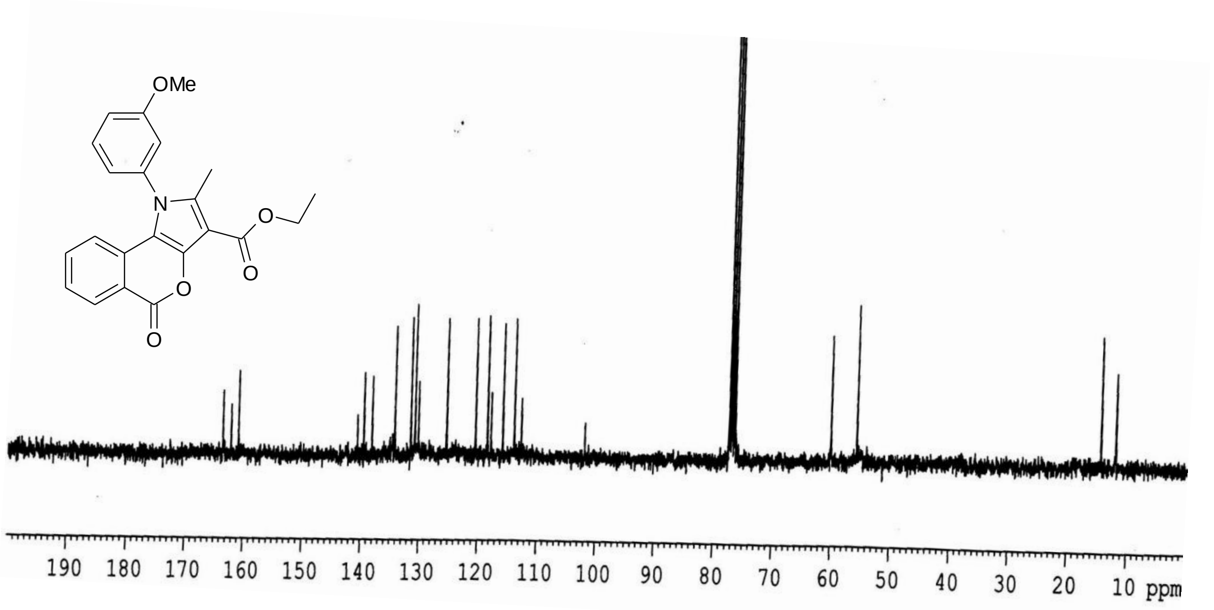

^{13}C NMR of Compound **8g** in $CDCl_3$

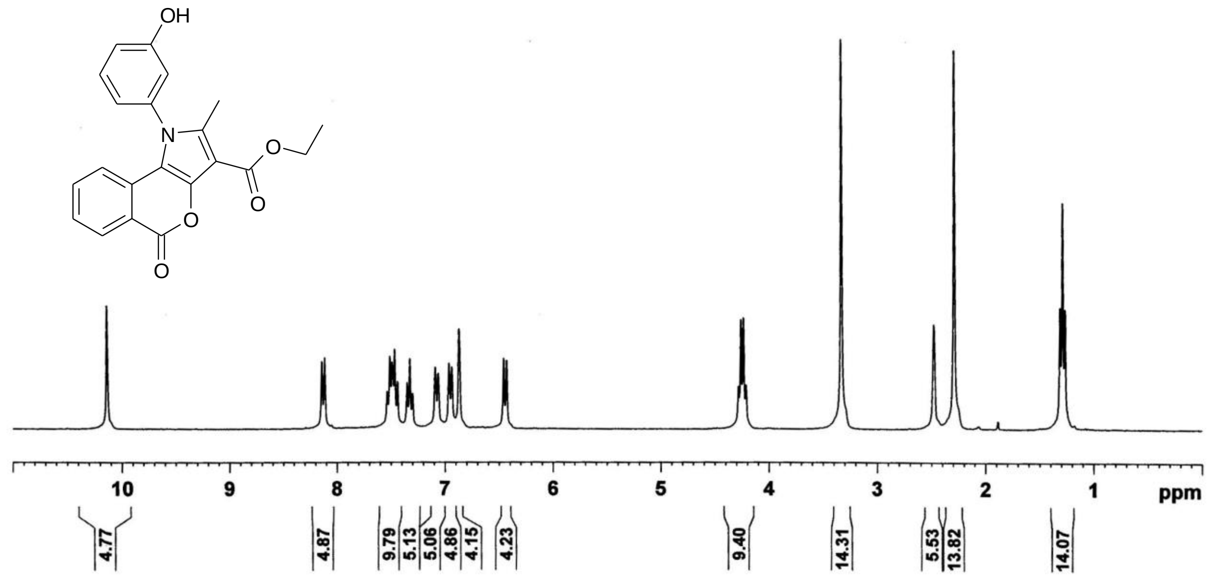

^{1}H NMR of Compound **8h** in d_6-DMSO

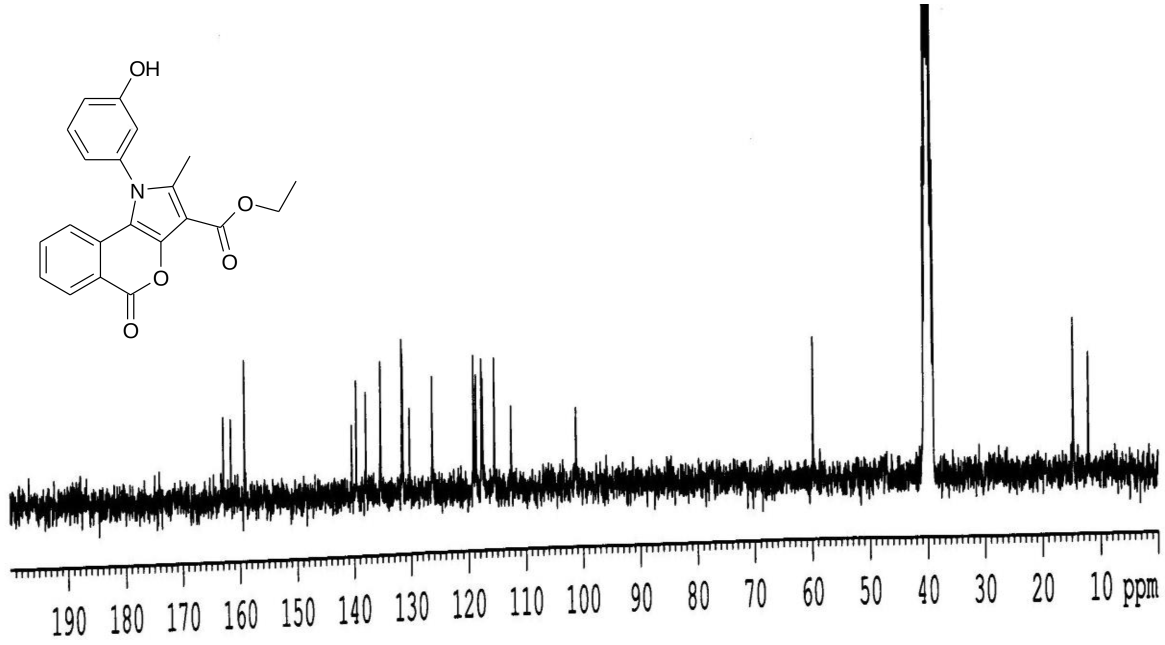

^{13}C NMR of Compound **8h** in d_6-DMSO

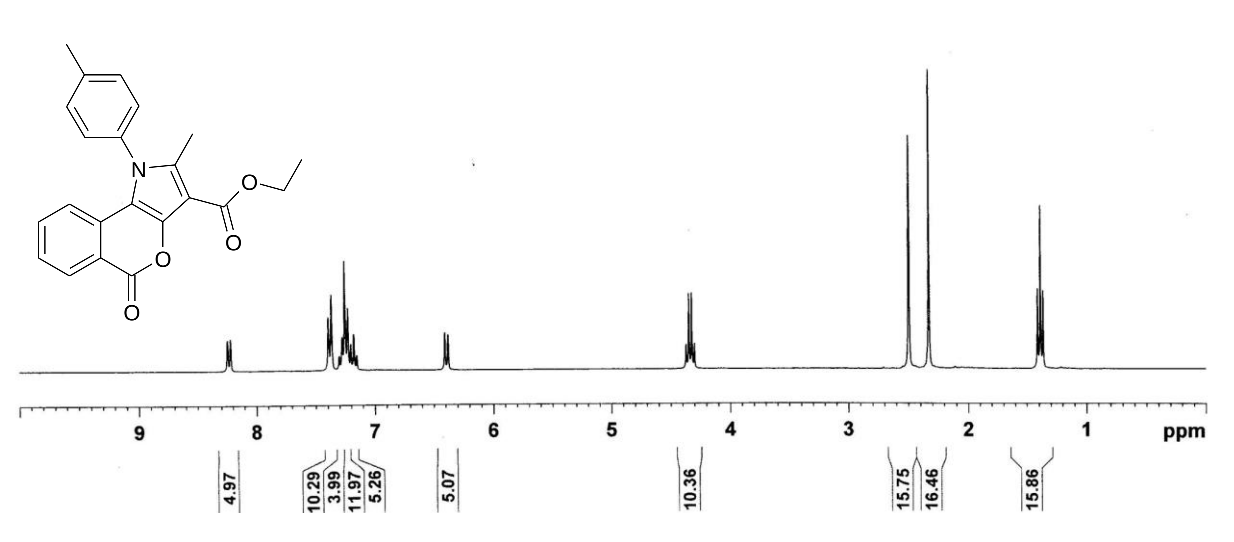

^{1}H NMR of Compound **8i** in $CDCl_3$

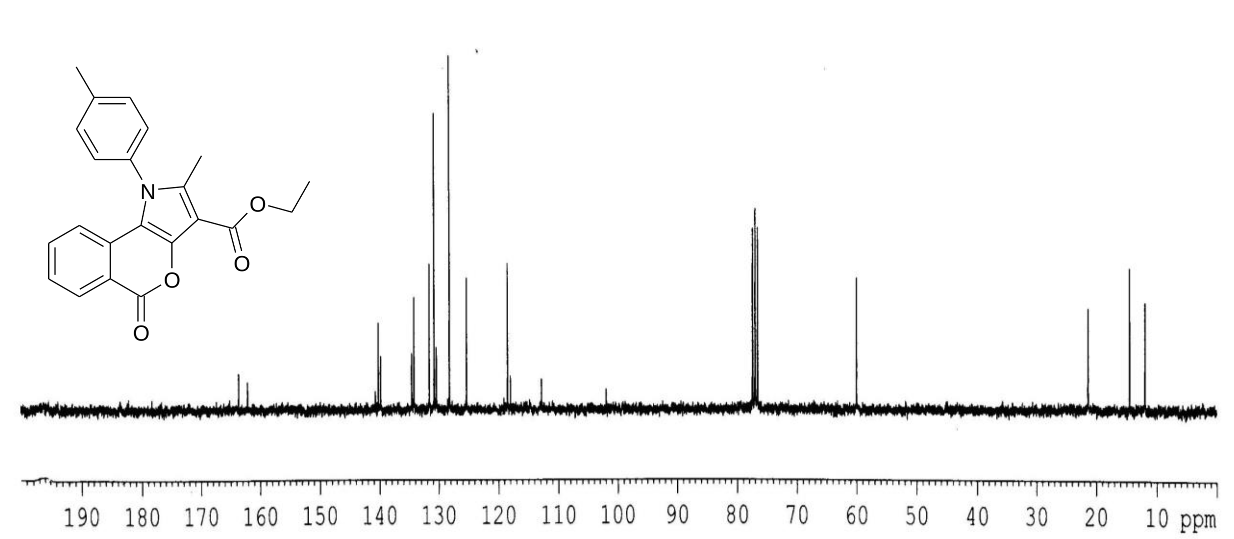

^{13}C NMR of Compound **8i** in $CDCl_3$

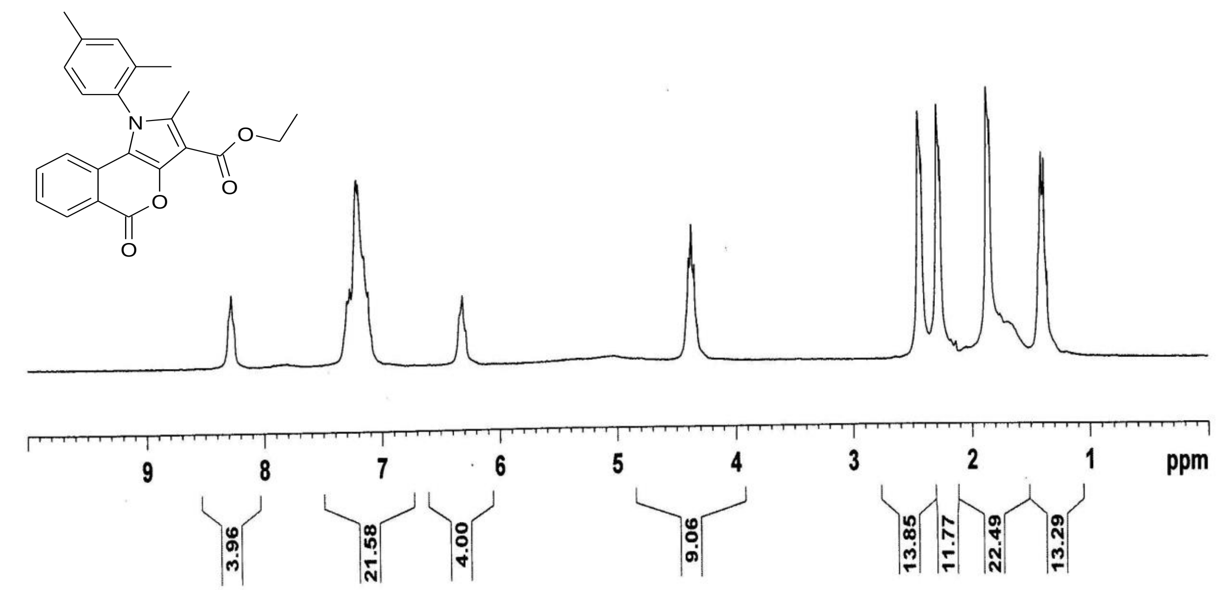

^{1}H NMR of Compound **8j** in $CDCl_3$

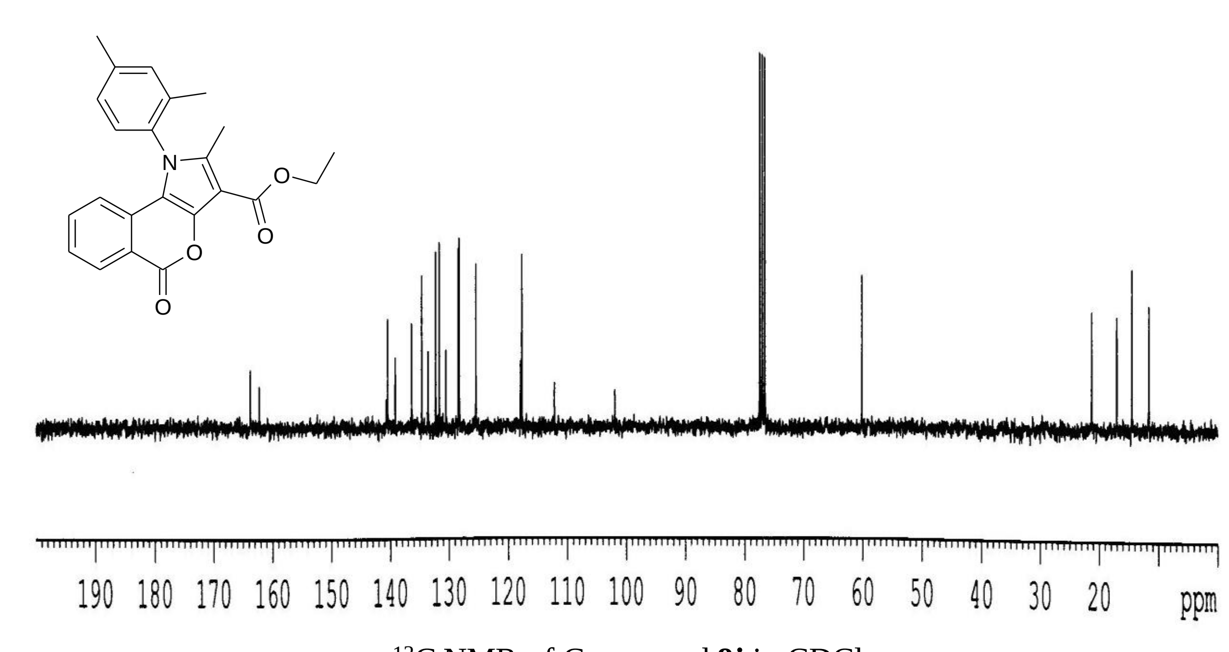

^{13}C NMR of Compound **8j** in $CDCl_3$

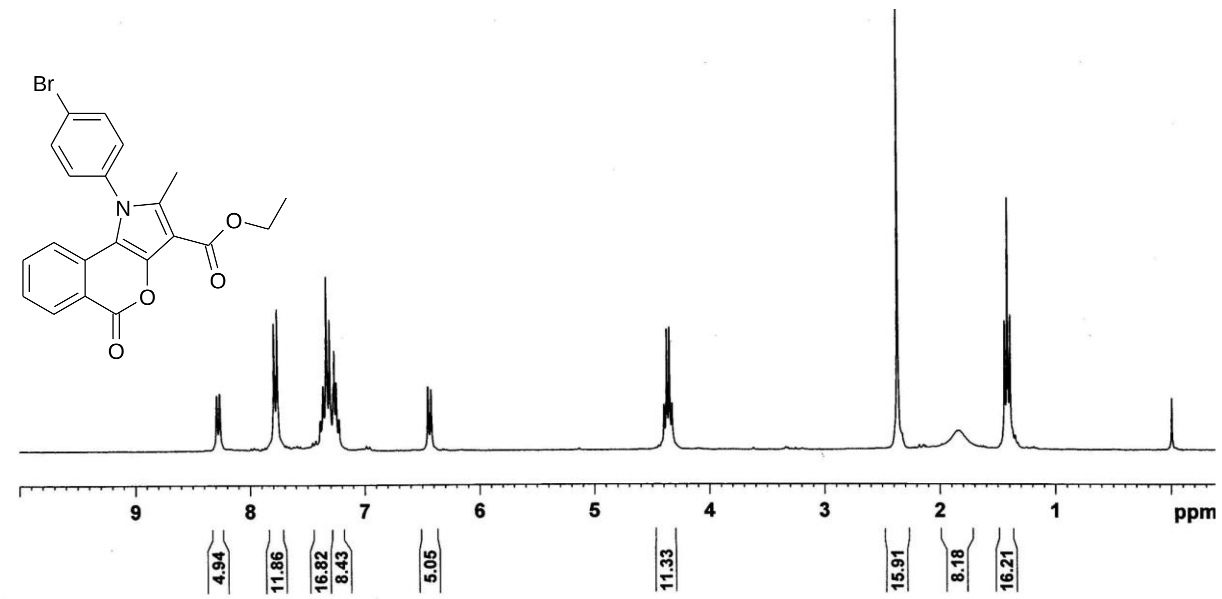

^{1}H NMR of Compound **8k** in $CDCl_3$

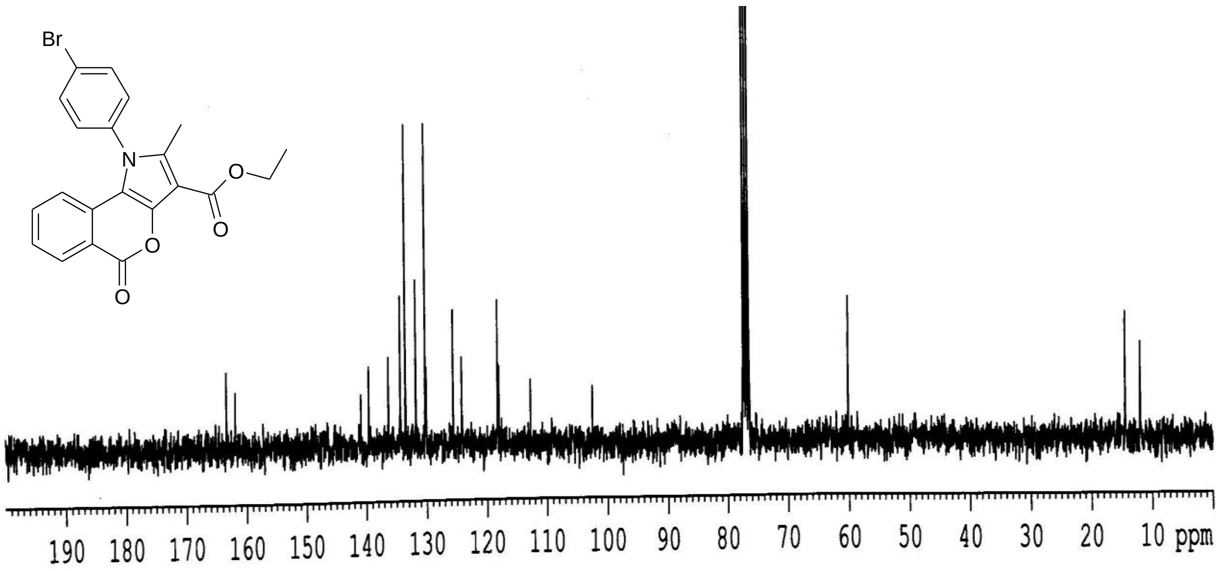

^{13}C NMR of Compound **8k** in $CDCl_3$

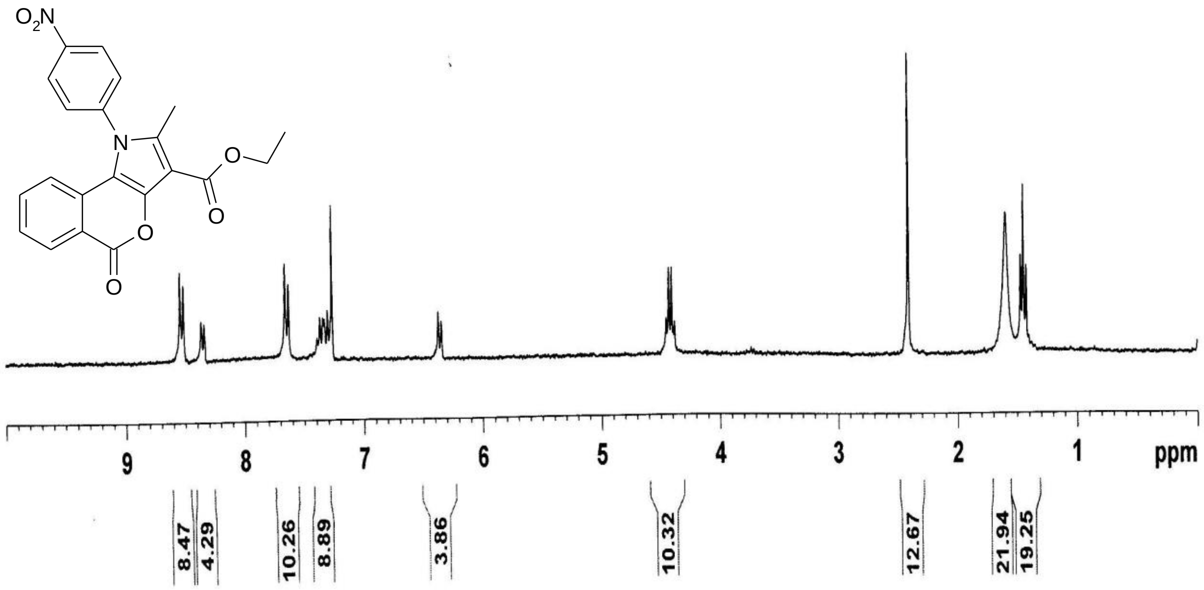

^{1}H NMR of Compound **8l** in $CDCl_3$

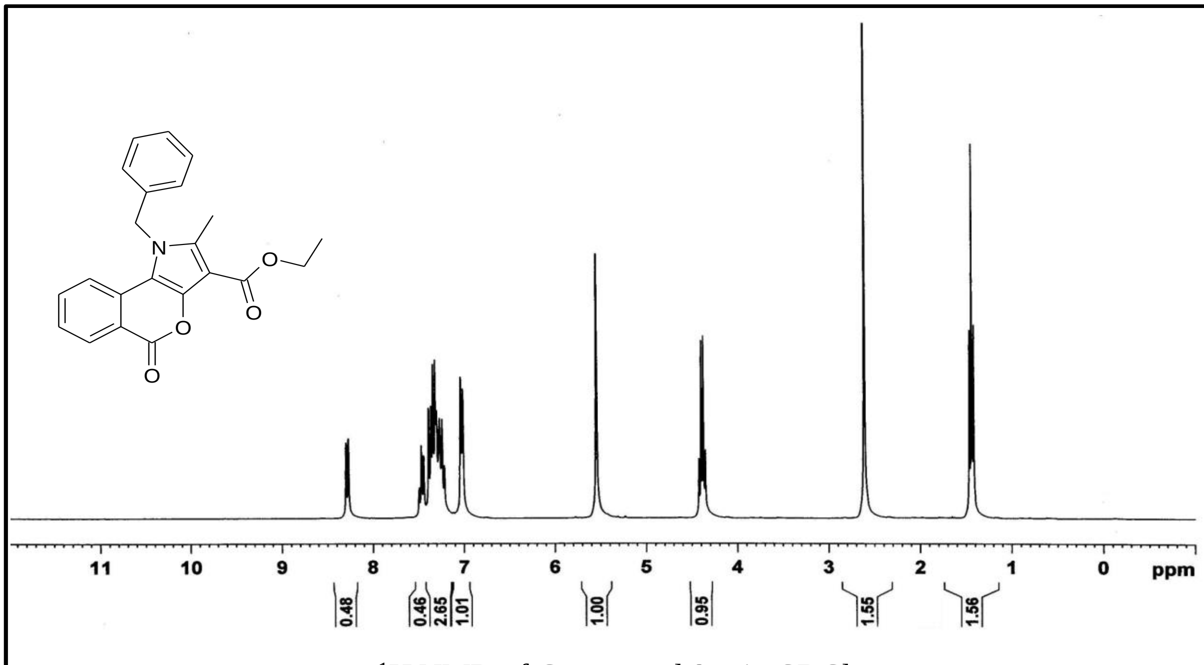

^{1}H NMR of Compound **8m** in $CDCl_3$

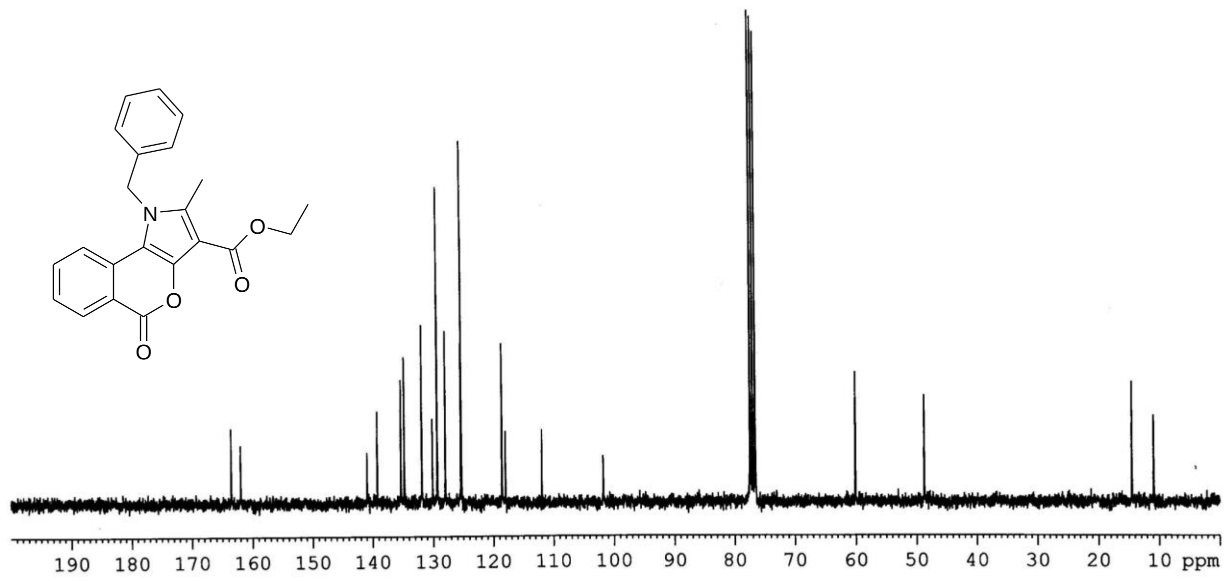

^{13}C NMR of Compound **8m** in $CDCl_3$

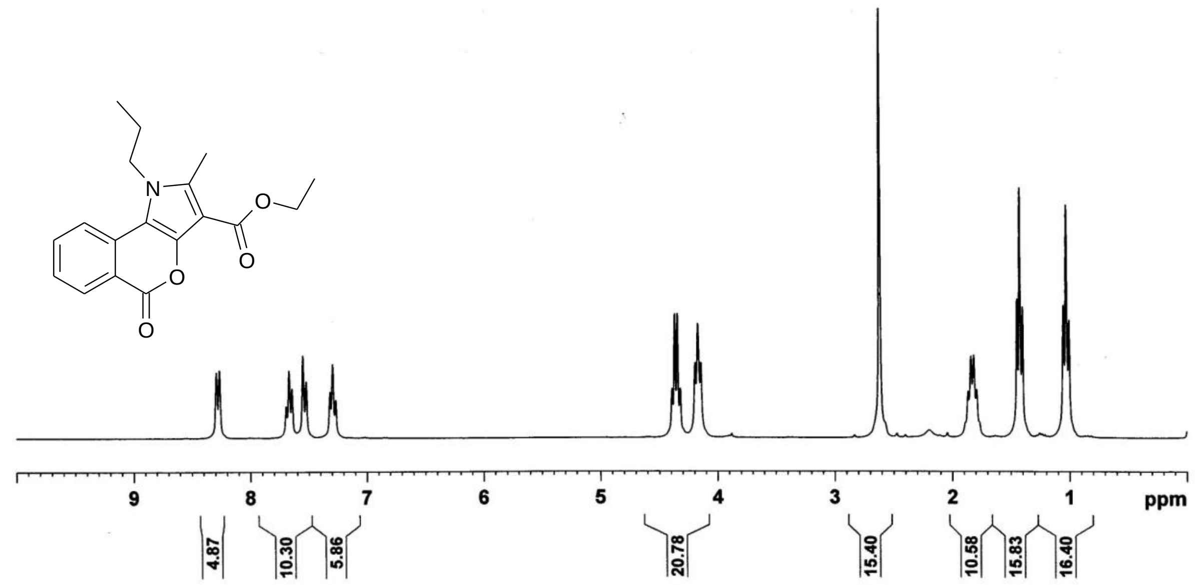

^{1}H NMR of Compound **8n** in $CDCl_3$

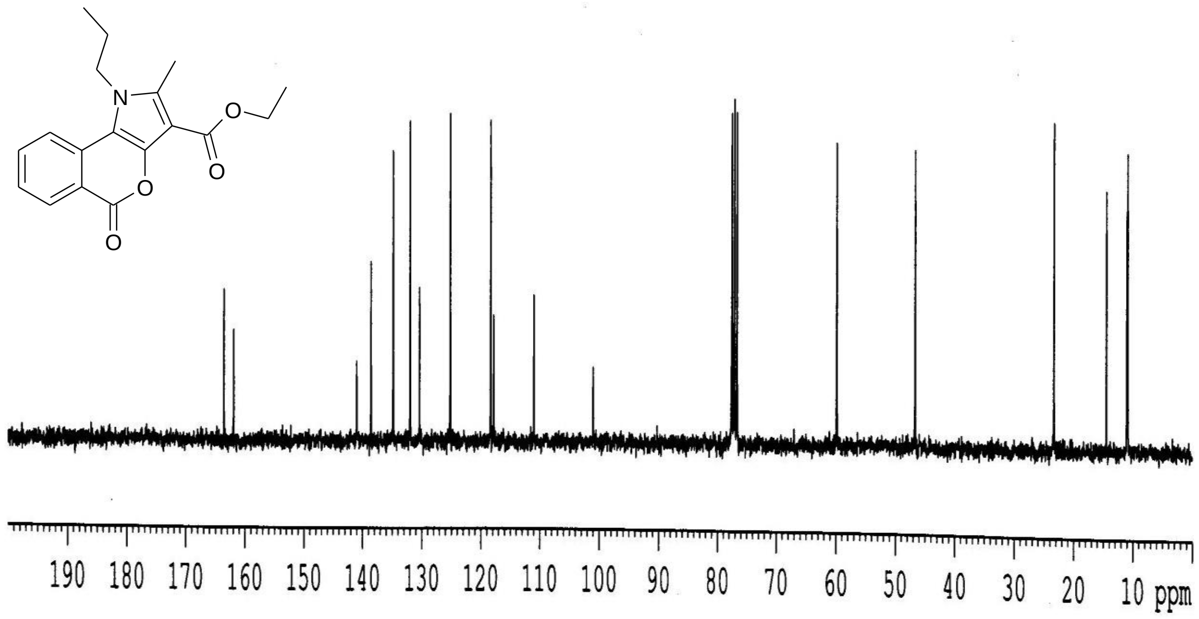

^{13}C NMR of Compound **8n** in $CDCl_3$

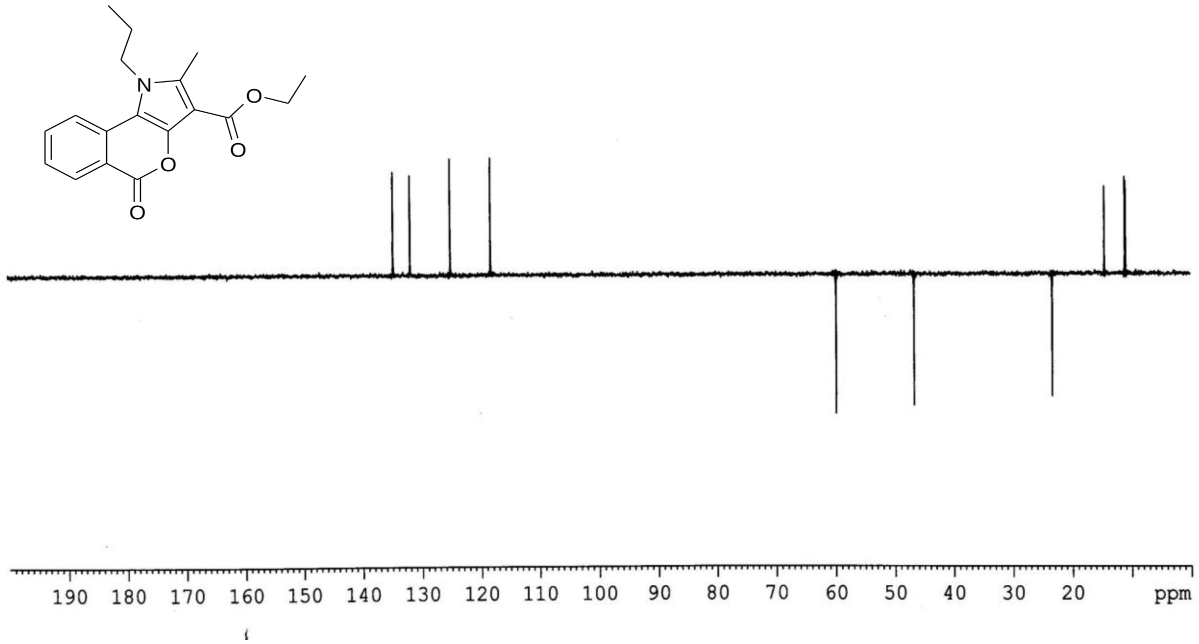

DEPT 135 NMR of Compound **8n** in $CDCl_3$

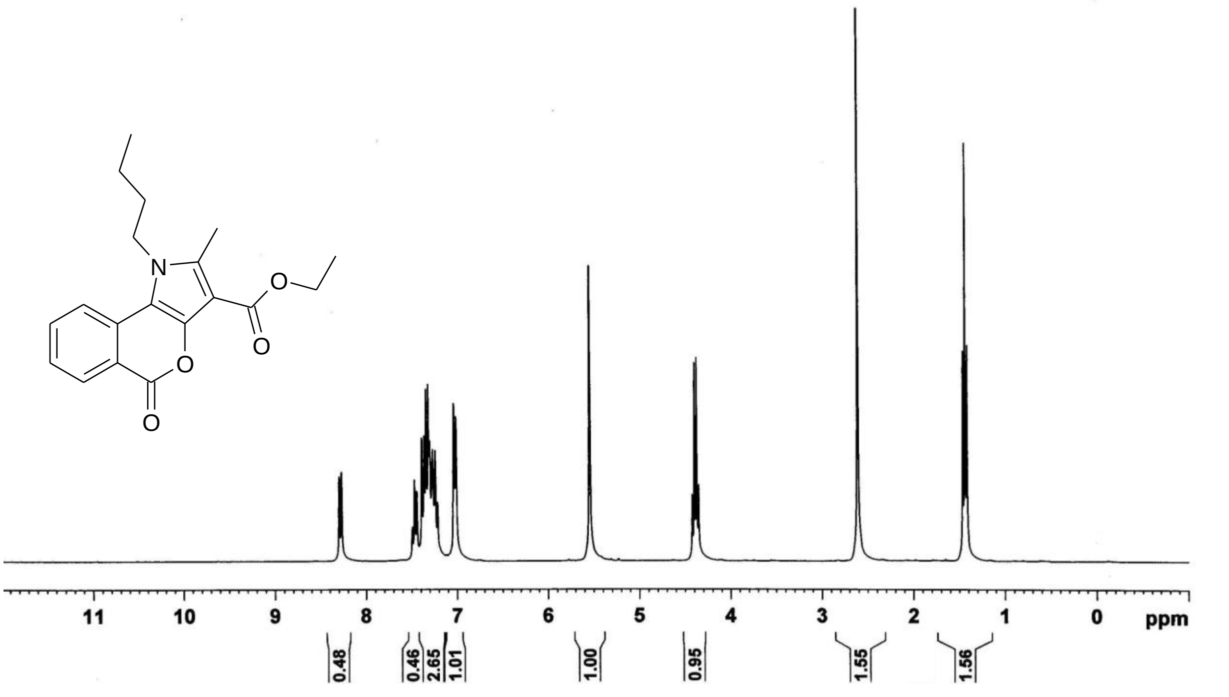

^{1}H NMR of Compound **8o** in $CDCl_3$

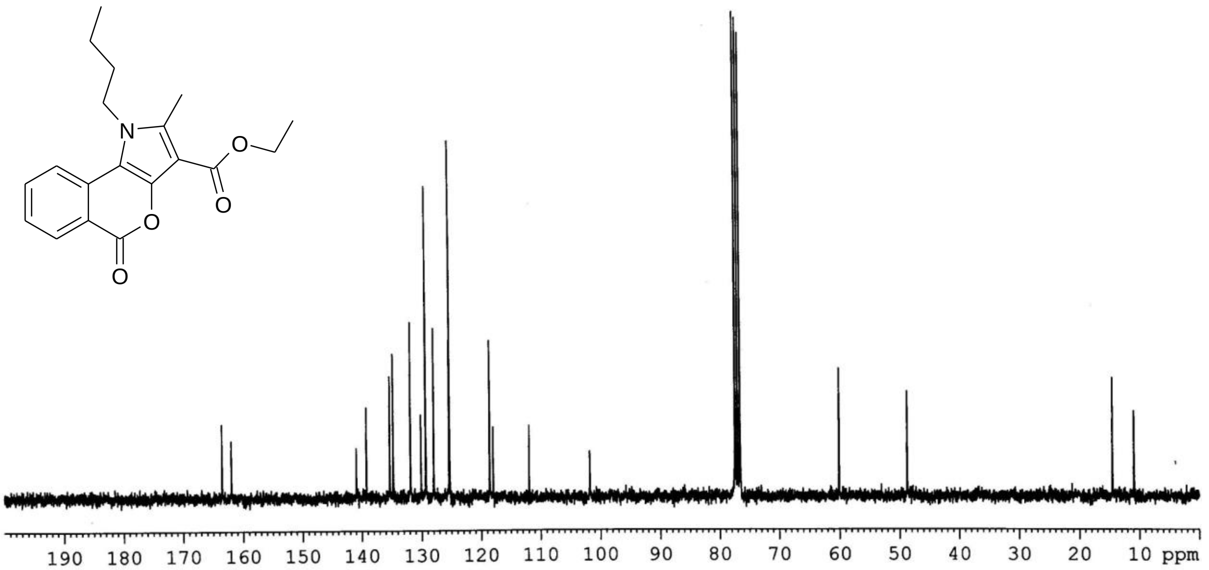

^{13}C NMR of Compound **8o** in $CDCl_3$
